# Whole-genome sequencing and antimicrobial potential of bacteria isolated from Polish honey

**DOI:** 10.1007/s00253-023-12732-9

**Published:** 2023-09-04

**Authors:** Ahmer Bin Hafeez, Karolina Pełka, Kamila Buzun, Randy Worobo, Piotr Szweda

**Affiliations:** 1 Department of Pharmaceutical Technology and Biochemistry, Faculty of Chemistry, Gdańsk University of Technology, Ul. G. Narutowicza 11/12, 80-233 Gdańsk, Poland; 2 Department of Biotechnology, Faculty of Pharmacy, Medical University of Bialystok, 15-089 Bialystok, Poland; 3 Department of Food Science, Cornell University, Ithaca, NY 14853 USA

**Keywords:** Honey, Whole-genome sequencing, *Bacillus* spp., Antimicrobial peptides

## Abstract

**Abstract:**

The aim of this study was the whole-genome analysis and assessment of the antimicrobial potential of bacterial isolates from honey harvested in one geographical location—the north of Poland. In total, 132 strains were derived from three honey samples, and the antimicrobial activity of CFAM (cell-free after-culture medium) was used as a criterion for strain selection and detailed genomic investigation. Two of the tested isolates (SZA14 and SZA16) were classified as *Bacillus paralicheniformis*, and one isolate (SZB3) as *Bacillus subtilis* based on their ANI and phylogenetic analysis relatedness. The isolates SZA14 and SZA16 were harvested from the same honey sample with a nucleotide identity of 98.96%. All three isolates have been found to be potential producers of different antimicrobial compounds. The secondary metabolite genome mining pipeline (antiSMASH) identified 14 gene cluster coding for non-ribosomal peptide synthetases (NRPs), polyketide synthases (PKSs), and ribosomally synthesized and post-translationally modified peptides (RiPPs) that are potential sources of novel antibacterials. The BAGEL4 analysis revealed the presence of nine putative gene clusters of interest in the isolates SZA14 and SZA16 (including the presence of six similar clusters present in both isolates, coding for the production of enterocin Nkr-5-3B, haloduracin-alpha, sonorensin, bottromycin, comX2, and lasso peptide), and four in *B. subtilis* isolate SZB3 (competence factor, sporulation-killing factor, subtilosin A, and sactipeptides). The outcomes of this study confirm that honey-derived *Bacillus* spp. strains can be considered potential producers of a broad spectrum of antimicrobial agents.

**Key points:**

*• Bacteria of the genus Bacillus are an important component of honey microbiota.*

*• Honey-derived Bacillus spp. strains are potential producers of new antimicrobials.*

**Supplementary Information:**

The online version contains supplementary material available at 10.1007/s00253-023-12732-9.

## Introduction

Bee products such as honey and propolis have been frequently used as traditional remedies since ancient times (Waddington 1991). It has been used by the Egyptians, Romans, Greeks, and Chinese for centuries for healing wounds, gut diseases, gastric ulcers, coughs, and sore throats (Al-Jabri 2013). Natural honey is a mixture of carbohydrates (82.4%), fructose (38.5%), glucose (31%), other sugars (12.9%), water (17.1%), protein (0.5%), and several minerals, vitamins, amino acids, and phenols, as well as traces of bioactive components such as phenolic acid, flavonoids, and α-tocopherol (Pasupuleti et al. 2017). The health benefits of honey are attributed to its constituents, which include phenolic acids, flavonoids, ascorbic acid, proteins, carotenoids, and certain enzymes such as glucose oxidase and catalase (Moniruzzaman et al. 2011). The wide range of antimicrobial activity of honey is due to various components contributing to its antibacterial potential: the sugar content, polyphenol compounds, hydrogen peroxide, 1,2-dicarbonyl compounds, and bee defensin-1 (Almasaudi 2021).

Raw honey is a reservoir for several microbial species like mold, yeast, and spore-forming bacteria (Snowdon and Cliver 1996). The bees collect nectar and pollen at a distance up to 2 km from the hive, which results in a high diversity of microorganisms in these “raw materials” (Brudzynski 2021). However, the diversity and composition of raw product microbiota change significantly as a result of the transformation of nectar to honey and bee pollen to bee bread. The majority of bacteria (including pathogenic species) are eliminated as a result of water evaporation (increased sugar concentration), acidification, and the presence of bee-derived antimicrobial compounds, e.g., bee defensin-1 and glucose oxidase and also antagonistic or competitive microbe-microbe interactions (Brudzynski 2021). Although the composition of the microbiota in bee products might differ with respect to botanical and geographical origins, the keystone species in the core microbiota of honey and bee bread are *Bacillus* and *Lactobacillus* (Grabowski and Klein 2017; Brudzynski 2021). Recent studies have shown that most of the LAB and *Bacillus* species, including *Lactobacillus casei*, *Lactobacillus plantarum*, *B. subtilis*, and *B. velezensis*, have been shown to exhibit antibacterial and antifungal activity and are promising sources of novel antimicrobials (Schnürer and Magnusson 2005; Lee et al. 2008b, a; Pajor et al. 2018; Wadi 2022; Xiong et al. 2022). *Bacillus* species are ubiquitous and survive in complex, competitive microbial communities like soil and the ocean (Harwood et al. 2018). Therefore, a large portion of their genome undergoes secondary metabolism and synthesizes diverse secondary metabolites exhibiting broad-spectrum antimicrobial activity (Motta et al. 2008; Li et al. 2012; Sabaté and Audisio 2013). Moreover, due to their spore-forming ability, they can withstand the harsh conditions of honey.

To date, the majority of microbial strains producing efficient antibiotics are recovered from soil (Newman and Cragg 2007). Several research groups, including ours, have recently revealed that honey (Lee et al. 2008b; Pajor et al. 2018, 2020; Xiong et al. 2022) and other bee products, such as bee pollen or bee bread (Didaras et al. 2020; Pełka et al. 2021a, b), can be considered a reservoir of microbes producing metabolites with antibacterial and antifungal potential. A majority of isolates derived from bee products exhibit antimicrobial (antibacterial and/or antifungal) potential (Lee et al. 2008b; Pajor et al. 2018; Pełka et al. 2021b), which is an important benefit of this reservoir. Both *Bacillus* and *Lactobacillus* are well-known producers of a broad spectrum of antimicrobial compounds, including antibiotics, bacteriocins, and biosurfactants (Zacharof and Lovitt 2012; Sumi et al. 2015). In this study, we performed whole-genome sequencing analysis and screening of secondary metabolites of *Bacillus* isolates to provide a better understanding of the molecular mechanism for this adaptation that might lead to the discovery of new antibacterial compounds.

## Methodology

### *Honey samples and isolation of bacterial strains*

The three samples of multi-flower honey (SZA, SZB, and SZC) were collected from two apiaries located in the Sulmin village (Pomeranian Voivodeship; North of Poland) on June 20, 2018, and the strains were isolated in October 2018. The honey samples were stored in the dark at ambient temperature. The isolation of bacterial isolates and determination of their antibacterial potential were performed according to the methodology presented in our previous publication (Pajor et al. 2018), with slight modifications. Briefly, the honey samples were diluted in sterile distilled water in a 1:1 (*v/v*) ratio, and 1 ml of each suspension was streaked on a Petri dish (*φ* = 200 mm) containing solid growth medium (Luria–Bertani—LB agar). The Petri dishes were then incubated overnight at 37 °C, and the growing colonies were counted.

Determination of antimicrobial potential (ability to produce antimicrobial metabolites) was performed by the transfer of selected colonies of bacteria with a sterile pipette tip on the surface of LB agar medium (in the form of about 10-mm-long lines) inoculated with *Staphylococcus aureus* ATCC 25923 or *S. aureus* ATCC 29213 strains. The pre-inoculation was performed by streaking *S. aureus* (the final optical density of each suspension was OD600 = 0.1) reference strains with a sterile cotton swab soaked in a suspension. Thereafter, the growth inhibition zones of *S. aureus* ATCC 25923 and *S. aureus* ATCC 29213 near the lines of the growing cells of the isolated bacteria were observed.

Furthermore, the selection of the most promising producing strains (PS—isolates that exhibited abilities to produce antimicrobial compounds) was performed by growing bacteria in the liquid (LB broth) medium and determining the presence of the active compounds in the cell-free after-culture medium (CFAM). The isolates were grown for 18–24 h (37 °C with shaking at 180 rpm). The CFAM was obtained by centrifugation (12,000 rpm, 15 min) and heat treatment of the supernatant (60 °C, for 10 min). A total of 50 µl of the CFAM was loaded into the wells on the Chapman agar plates, which were already inoculated with the *S. aureus* reference strain. The plates were incubated for 18–24 h at 37 °C and growth inhibition zones were observed and measured.

### DNA isolation and sequencing

Isolation and sequencing of bacterial genomic DNA were performed in the DNA Sequencing and Oligonucleotide Synthesis Laboratory of the Institute of Biochemistry and Biophysics, Polish Academy of Science (Warsaw, Poland). Cell pellets from the overnight LB culture of the isolates were treated with the CTAB/lysozyme method and the template DNA quality and quantity were checked on the agarose gel. Genomic bacterial DNA was mechanically sheared to an appropriate size for Paired-End TruSeq-like library construction using the KAPA Library Preparation Kit (KAPA/Roche, Basel, Switzerland) following the manufacturer’s instructions. The bacterial genomes were sequenced in paired-end mode (v3, 600 cycle chemistry kit) using MiSeq (Illumina, San Diego, CA).

A quality check for raw reads was performed with FASTQC and reads were trimmed and paired using Trimmomatic (version 0.38.0) (Bolger et al. 2014) with the following parameters: LEADING: 3 TRAILING: 3 SLIDINGWINDOW: 4:20 MINLEN: 25. A secondary read quality check was performed after trimming to ensure normal results for “per base sequence quality,” “per base N content,” “sequence duplication levels,” and “adapter content.” De novo assembly was performed with Unicycler (version 0.4.8.0) (Wick et al. 2017) using default k-mer settings for bacterial genome assembly. Scaffolds less than 500 bp were removed and assembly statistics [e.g., number of contigs, N50 (widely used to assess the contiguity of an assembly), G + C content] were assessed using QUAST (Version 5.0.2) (Gurevich et al. 2013). The resulting final assembly files were BLASTed in the National Center for Biotechnology Information (NCBI) and the genome assemblies of closely related group-type strains were downloaded from the assembly database. Average nucleotide identity (ANI) of the isolates was calculated with the OrthoANI method using OAT (version 0.93.1) and BLAST + (version 2.13.0) (Lee et al. 2016). Rapid genome annotation was performed using Prokka (version 1.14.6) (Seemann 2014). Additional genome annotation was performed with the NCBI Prokaryotic Genome Annotation Pipeline (PGAP) (https://github.com/ncbi/pgap) on a local machine (Tatusova et al. 2016). Assembled genomes of *B. paralicheniformis*_SZA14, *B. paralichenifromis*_SZA16, and *B. subtilis*_SZB3 were submitted to Sequence Read Archive (SRA) and GenBank under the BioProject IDs PRJNA925310, PRJNA926019 and PRJNA926020, respectively. SRA accession numbers for *B. paralicheniformis*_SZA14, *B. paralicheniformis*_SZA16, and *B. subtilis*_SZB3 are SRR23118077, SRR23175314, and SRR2315315, respectively. Moreover, the selected strains were deposited to the German Collection of Microorganisms and Cell Cultures (DSMZ, https://www.dsmz.de/collection/deposit/open-collection/microorganisms) under the numbers DSM 115813; *B. paralicheniformis*_SZA14, DSM 115814; *B. paralicheniformis*_SZA16 and DSM 115815; *B. subtilis*_SZB3. The functional annotations were carried out using the eggNOG mapper v2 (http://eggnog-mapper.embl.de/) (Cantalapiedra et al. 2021).

### Whole-genome-based phylogenetic analysis

The whole-genome-based taxonomic analysis was performed by the Type (strain) Genome Server (TYGS) (https://tygs.dsmz.De) (Meier-Kolthoff 2019). The draft genomes of isolates SZA14, SZA16 and SZB3 sequenced in this study and 17 other closely related genomes of the *B. paralicheniformis* and *B. subtilis* groups, as well as the complete genome of *Bacillus capparidis* strain DSM103394 as an outgroup, were extracted from NCBI (accession numbers Supplementary Table S1) and submitted to the TYGS server, settings: restricted genome mode. A phylogenomic tree was constructed with FastME (based on balanced minimum evolution and renders distance algorithms to infer phylogenies) (Lefort et al. 2015) using the genome blast distance phylogeny (GBDP) method and annotated using Interactive Tree of Life (iTOL) v5, an online tool for phylogenetic tree display and annotation (Letunic and Bork 2021). All pair-wise genome comparisons were carried out with GBDP and inter-genomic distances inferred under the algorithm “trimming” and distance formula d5 (Meier-Kolthoff 2019). The tree was rooted at the midpoint (Farris 1972). Branch supports were inferred from 100 pseudo-bootstrap replicates.

### Core SNP–based phylogeny

For further validation, a single nucleotide polymorphism (SNP)–based tree was constructed using the program kSNP v4.0. The optimal k-mer size of 17 was determined using the Kchooser module of kSNP v4.0 (Gardner and Hall 2013). The phylogenetic analysis was performed with core SNPs using a Bayesian approach as implemented in MrBayes version 3.2.6 (available on http://www.phylogeny.fr/one_task.cgi?task_type=mrbayes) (Ronquist et al. 2012). Under the likelihood model, the substitution types were set to General Time-Reversible 6 (GTR), the substitution model was kept as the default, and variation rates across sites were selected as invariable + gamma. Markov chain Monte Carlo parameters, i.e., the number of generations, were 100,000. The tree was sampled every 1000 generations and the first 100 trees sampled were discarded. The tree was inferred with iTOL v5 (Letunic and Bork 2021).

### A broad analysis of the genomes using genome mining tools

The genomes of isolates SZA14, SZA16, and SZB3 were compared to the respective type strain with Mauve (v20150226) using progressive alignment and seed-families options (Darling et al. 2004). The secondary metabolite genome mining pipeline (antiSMASH), with parameter strict, was used for the identification of gene clusters coding for potential secondary metabolites (Blin et al. 2021). Putative bacteriocin genes were detected with BAGEL4 (Van Heel et al. 2018). The web server Prophage hunter was employed for rapid identification and annotation of prophage sequences within bacterial genomes (Song et al. 2019).

### Functional annotation of the carbohydrate-active enzyme (CAZyme)

Accurate functional annotation of the proteome is the cornerstone of a successful genomics project. Different carbohydrate-active enzymes (CAZymes) were annotated by the dbCAN database (https://bcb.unl.edu/dbCAN2/index.php), a Meta server for automated carbohydrate-active enzyme annotation which were then summarized after manual curation and prospected using the Enzyme Commission (EC) number (Zhang et al. 2018). The biotechnological applications were determined by the list of the Association of Manufacturers and Formulators of Enzyme Products (AMFEP) and BRENDA database (http://www.brenda-enzymes.org/) (Placzek et al. 2017).

### Identification of antimicrobial peptides

For the detection of peptides with antimicrobial potential, online antimicrobial peptide database servers AMPA (https://tcofee.crg.cat/apps/ampa/do) (Torrent et al. 2012), ADAM (https://bioinformatics.cs.ntou.edu.tw/ADAM/) (Lee et al. 2015) and CAMPR4 (http://www.camp.bicnirrh.res.in/) (Waghu et al. 2016) were used. Commonly predicted peptides with lengths between 5 and 150 residues were analyzed sequentially in the APD3 database. The criteria for a positive conclusion for possible antimicrobial activity prediction in AMPA, ADAM, and CAMPR3 were defined as > 0.15 in AMPA, > 1 in ADAM, and > 0.5 in CAMPR3 (Pavlova et al. 2020).

## Results

### Strain selection

Considering the level of microbial contamination of the honey samples, some important differences were observed. The highest number of colonies (*n* = 116) was grown on the plate inoculated with the suspension of honey A, while only 12 and 4 colonies were grown on the plates that were inoculated with honey samples B and C, respectively.

The screening for the production of antimicrobial compounds was performed on randomly selected 20, 10, and 3 colonies recovered from honey samples A, B, and C, respectively. Anti-staphylococcal activity and the presence of *Staphylococci* growth inhibition zones around the colonies of bacterial isolates were identified for 7 out of 20 isolates from product A (35%), 5 out of 10 (50%) from product B, and 2 out of 3 (66%) from product C. However, further analysis confirmed the presence of antimicrobial metabolites in the CFAM for 7 strains isolated from sample A, 2 strains isolated from sample B, and none from sample C.

**Fig. 1:**
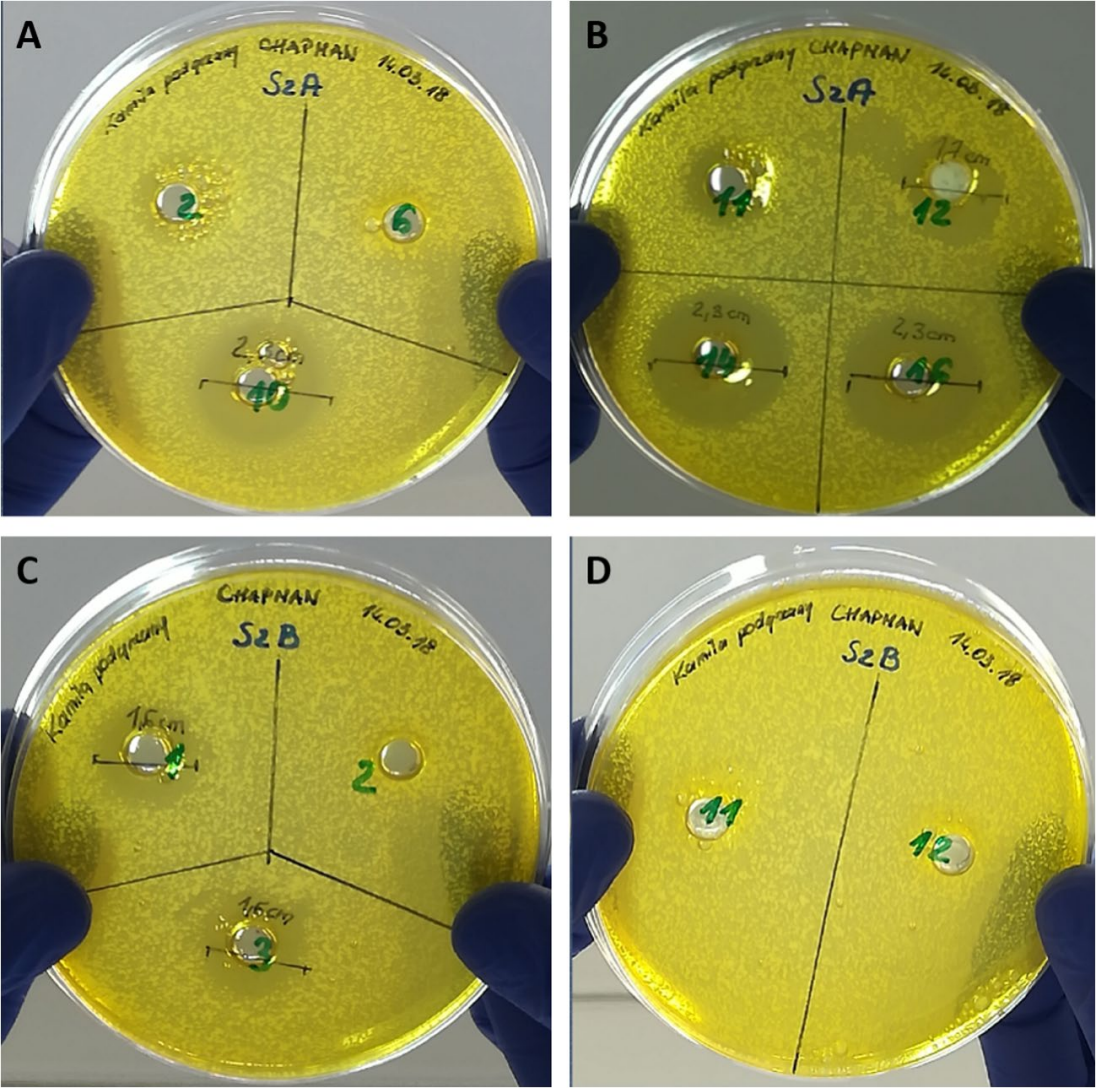
Anti-staphylococcal activity of CFAM **(**cell-free after-culture medium) of selected strains isolated from honey SZA (**A** and **B**) and SZB (**C** and **D**)

The bacterial isolates from the sample, SZA14, SZA16, and SZB3 (Fig. 1), were potentially the best producers of antimicrobials and were selected for whole-genome sequencing, aiming to identify the genes responsible for the synthesis of antimicrobial compounds.

### Whole-genome sequencing and genome analysis

The 4,507,369 bp genome of isolate SZA14 was assembled into 46 contigs with a GC content of 45.59% and an N50 of 276,250 bp (Supplementary Table S2, Quast report). The 4,379,317 bp genome of isolate SZA16 was assembled into 21 contigs with a GC content of 45.81% and an N50 of 668,104 bp (Supplementary Table S2, Quast report). The genome of isolate SZB3 (4,244,057 bp) was assembled into 19 contigs with a GC content of 43.42% and an N50 of 2,257,132 (Supplementary Table S2, Quast report). Isolate SZA14 contained an estimated 4627 genes and 4541 coding sequences (CDSs), 86 RNAs, 3 rRNAs (5S, 16S, and 23S), and 78 tRNAs. Isolate SzA16 consisted of an estimated 4439 genes, 4352 CDSs, 87 RNAs, 3 rRNAs (5S, 16S, and 23S), and 79 tRNAs. On the other hand, isolate SZB3 had 4489, 4408 CDSs, 81 RNAs, 3 rRNAs (5S, 16S, and 23S), and 73 tRNA.

Average nucleotide identity (ANI) classified the isolates at the species level. Isolates SZA14 and SZA16 were most closely related to type strains *B. paralicheniformis* HAS-1 and B*. paralicheniformis* FA6, with orthoANI values of 99.36% and 99.98%, respectively. Isolate SZB3 was closely related to *B. subtilis* strain P9B1 with an orthoANI value of 99.96% (Table 1). The ANI between isolates SZA14 and SZA16 was 98.96%. Based on the proposed species boundary of 95–96% orthoANI value, SZA14 and SZA16 were classified as *B. paralicheniformis* species and SZB3 as *B. subtilis* specie (Goris et al. 2007; Richter and Rosselló-Móra 2009).

**Table 1:** Average nucleotide identity (ANI) calculated by OAT with BLAST +

Query genome	Reference genome	OrthoANI value (%)
Isolate SZA14	*Bacillus paralicheniformis*_StrainHAS-1	99.3641
Isolate SZA14	*Bacillus paralicheniformis*_StrainTXO7B-1SG6	99.2902
Isolate SZA14	*Bacillus paralicheniformis*_StrainBL-09	98.9642
Isolate SZA14	*Bacillus paralicheniformis*_StrainNCTC8721	99.2516
Isolate SZA16	*Bacillus paralicheniformis*_FA6	99.9764
Isolate SZA16	*Bacillus paralicheniformis*_StrainCBMAI1303	99.9734
Isolate SZA16	*Bacillus paralicheniformis*_StrainJ36TS2	99.9504
Isolate SZA16	*Bacillus paralicheniformis*_ATCC9945a	99.9586
Isolate SZB3	*Bacillus subtilis*_StrainP9_B1	99.9623
Isolate SZB3	*Bacillus subtilis*_StrainBsi	99.8094
Isolate SZB3	*Bacillus subtilis*_StrainFJAT-4	99.841
Isolate SZB3	*Bacillus subtilis*_StrainP8_B3	99.8312

### Phylogenetic analysis

**Fig. 2:**
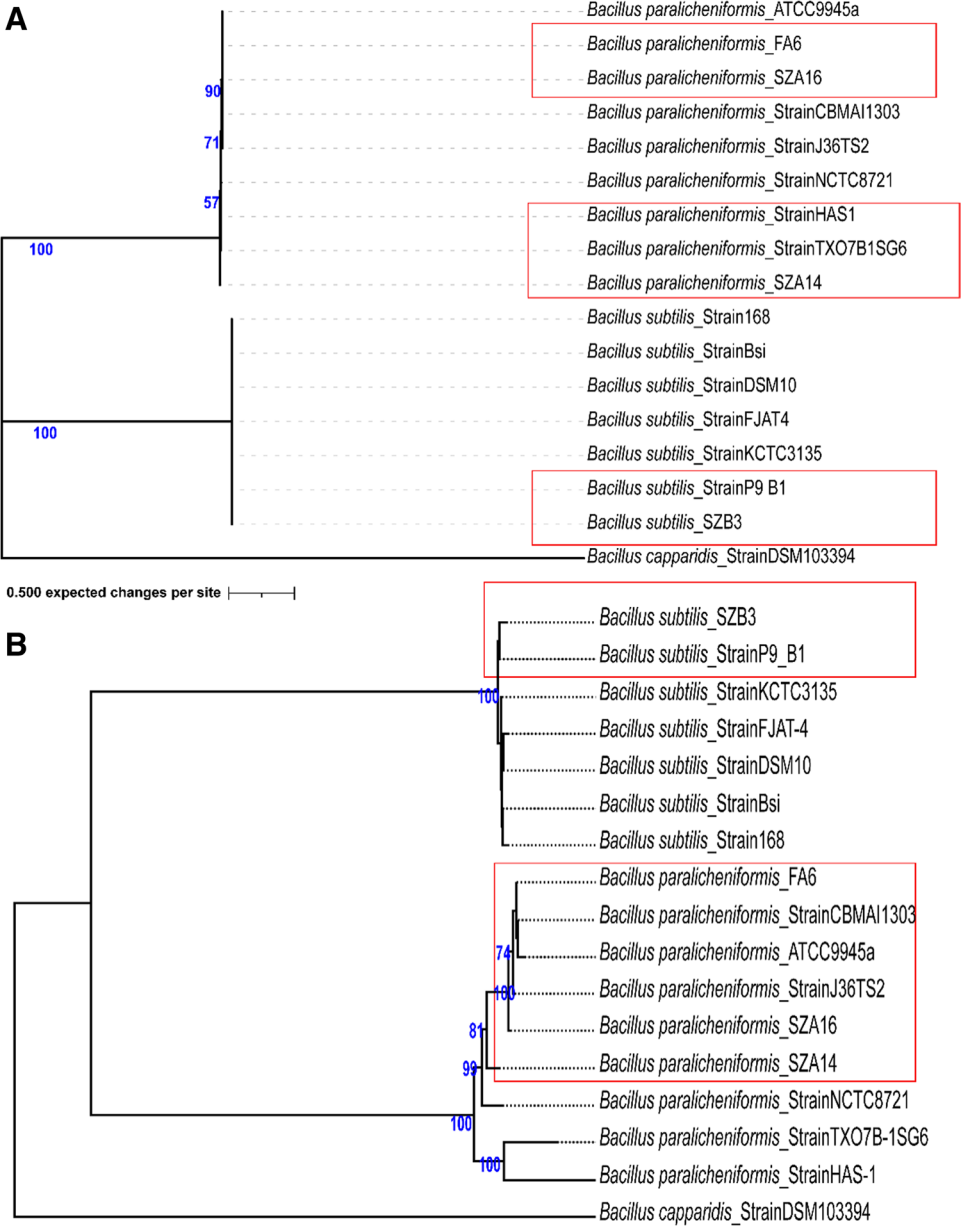
**A** Core SNP–based Bayesian phylogenetic tree inferred by MrBayes. The branch structure was confirmed by a bootstrap consensus tree inferred from 1000 replicates and annotated in iTOL. **B** Genome tree inferred with FastME 2.1.6.1 (Lefort et al. 2015) from GBDP distances calculated from genome sequences. The branch lengths are scaled in terms of the GBDP distance formula d5. The numbers above branches are GBDP pseudo-bootstrap support values > 60% from 100 replications, with an average branch support of 21.2%. The tree was rooted at the midpoint (Farris 1972). The trees were finally imported to Adobe Illustrator for refinement

To elucidate the phylogenetic relationships between our isolates and the closely related *Bacillus* species, a total of 17 genomes were downloaded from the NCBI database. Nine *B. paralicheniformis* group isolates, seven *B. subtilis* isolates, and one *B. capparidis* strain DSM103394 as an outgroup were included in the phylogenetic analysis. The phylogenetic tree based on the SNPs and the entire genome revealed that the isolates from this study have close relatedness to the type strains *B. paralicheniformis* (isolates SZA14 and SZA16) and *B. subtilis* (strain SZB3) (Fig. 2).

### Functional prediction

**Fig. 3:**
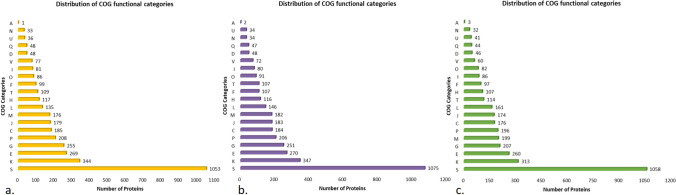
Distribution of Cluster of Orthologous group (COG) functional categories to the proteins of isolate **a** SZA14; **b** SZA16; **c** SZB3. The *X*-axis denotes the number of protein and *Y*-axis denotes COG categories. Each alphabet represents a unique COG category. A—RNA processing and modification; C denotes energy production and conversion; D—cell cycle control, cell division, and chromosome partitioning; E—amino acid transport and metabolism; F—nucleotide transport and metabolism; G—carbohydrate transport and metabolism; H—coenzyme transport and metabolism; I—lipid transport and metabolism; J—translation, ribosomal structure, and biogenesis; K—transcription; L—replication, recombination, and repair; cell wall/membrane/envelope biogenesis; N—cell motility; O—posttranslational modification, protein turnover, chaperones; P—inorganic ion transport and metabolism; Q—secondary metabolite biosynthesis, transport, and catabolism; S—function unknown; T—signal transduction mechanisms; U—intracellular trafficking, secretion, and vesicular transport; and V—defense mechanisms

Clusters of orthologous groups (COG) functional group analysis was performed using eggNOG mapper v2. For the isolate SZA14, 4105 genes out of 4462 (91%) were assigned to 21 clusters (Fig. 3). The Function unknown (S: 1075) category had the highest numbers, implicating the uniqueness and yet-to-explore potential of this isolate. The remaining proteins were categorized under functional groups such as transcription (K: 347); amino acid transport and metabolism (E: 270); carbohydrate transport and metabolism (G: 251); inorganic ion transport and metabolism (P: 206); energy production and conversion (C: 184); translation, ribosomal structure and biogenesis (J: 183); cell wall/membrane/envelope biogenesis (M: 182); replication, recombination and repair (L: 146); coenzyme transport and metabolism (H: 116); nucleotide transport and metabolism (F: 107); signal transduction mechanisms (T: 107); posttranslational modification, protein turnover, chaperones (O: 91); lipid transport and metabolism (I: 80); defense mechanisms (V: 72); chromosome partitioning (D: 48); cell cycle control, cell division, secondary metabolites biosynthesis, transport and catabolism (Q: 47); cell motility (N: 34) intracellular trafficking, secretion, and vesicular transport (U: 34); and RNA processing and modification (A: 2) (Fig. 3a). 4026 entries out of 4270 (94%) for strain SZA16 were classified into 21 different COG categories (3b) and for SZB3 3976 out of 4348 entries (91%) were assigned to 21 different COG categories (3c).

### BRIG and secondary metabolite analysis

**Fig. 4:**
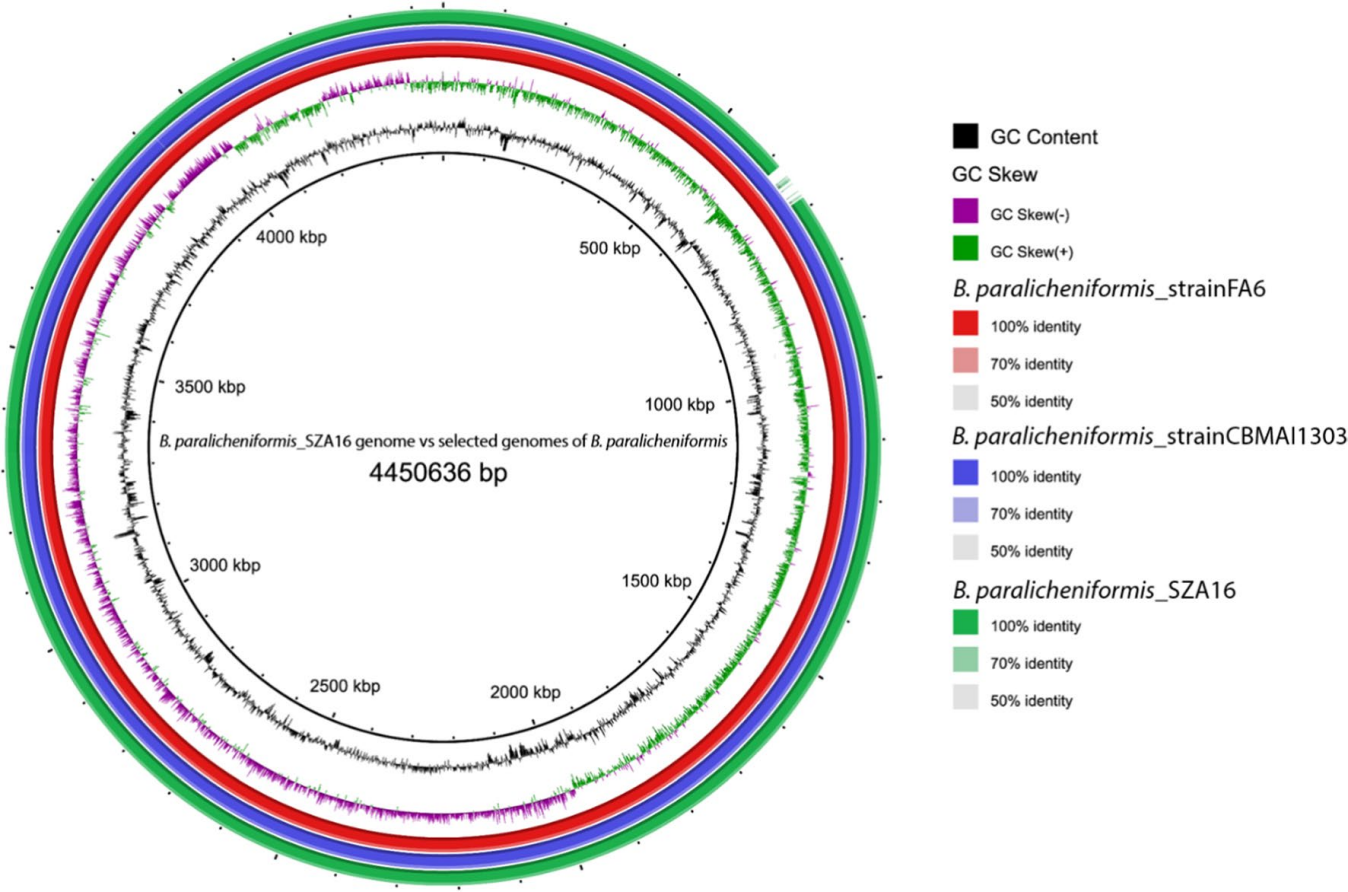
Genome comparison of *B. paralicheniformis*_SZA16 against closely related *Bacillus* type strains, *B. paralicheniformis*_strainFA6, and *B. paralicheniformis*_strainCBMAI1303. From the inner to the outer ring: (1) GC Content, black; (2) GC Skew, purple-green; (3) *B. paralicheniformis* strain FA6 nucleotide sequence, red; (4) *B. paralicheniformis*_strainCBMAI1303 nucleotide sequence, blue; (5) *B. paralicheniformis*_SZA16 nucleotide sequence, green. The circular ring map was constructed by the BLAST Ring Image Generator (BRIG, version 0.95)

Additional genome comparisons of *B. paralicheniformis* type strains and *B. subtilis* strains and isolates SZA14, SZA16, and SZB3 were visualized with BRIG version 0.95. The diagrammatic representation of SZA16 is represented in Fig. 4, while that of SZA14 and SZB3 is represented in (Supplementary Figures S1a and S1b). Gaps in the circular chromosome represented regions with no homology to the reference strains. Gaps for the isolates in our study were consistent due to high levels of nucleotide homology. Gaps at several positions observed when comparing isolates from this study with the closely related *Bacillus* strains indicated the potential presence of novel gene products. When visualized with Mauve, divergent regions and genetic rearrangement were observed, indicating the presence of mobile genetic elements (plasmids, prophages, and transposons) within each genome, specifically prophages.

AntiSMASH identified secondary metabolite biosynthetic gene clusters (BGCs), including non-ribosomal peptide synthetases (NRPs), polyketide synthases (PKSs), ribosomally synthesized and post-translationally modified peptides (RiPPs), and other antimicrobial synthases. A total of 14 putative BGCs were identified in the genome of isolate SZA14, including 6 NRPs for lichenysin, fengycin, bacillibactin, bacitracin, one type III PKS, and one Ripp cluster for lanthipeptide, thiopeptide, and others (Table 2). Fourteen putative BGCs were reported from the isolates SZA16 and SZB3, which are presented in Supplementary Table S3.

**Table 2:** List of identified secondary metabolite cluster of SZA14 using strictness “strict”

Region	Type	From	To	Most similar known cluster		Similarity
Region 1.1	NRP-metallophore, NRPS	179,782 bp	226,948 bp	Bacillibactin/bacillibactin E/bacillibactin F	NRP	100%
Region 1.2	Lasso peptide	329,843 bp	352,304 bp			
Region 2.1	RiPP-like	590, 754 bp	601,098 bp			
Region 3.1	Thiopeptide, RiPP-like	1 bp	37,353 bp	Butirosin A/butirosin B	Saccharide	7%
Region 3.2	NRPS-independent-siderophore	157, 466 bp	172, 929 bp	Schizokinen		
Region 6.1	NRPS	188, 517 bp	253, 960 bp	Lichenysin	NRP	100%
Region 7.1	NRPS, betalactone	143, 176 bp	181, 683 bp	Fengycin	NRP	60%
Region 8.1	NRPS	1 bp	21,785 bp	Fengycin	NRP	20%
Region 8.2	Terpene	11,337 bp	139,226 bp			
Region 9.1	NRPS	40,554 bp	123,443 bp	Bacitracin	NRP	100%
Region 11.1	T3PKS	63,325 bp	104,422			
Region 14.1	Lanthipeptide-class-ii, cyclic-lactone-autoinducer	1 bp	25,154 bp	Amyloliquecidin GF610	Ripp	93%
Region 23.1	CDPS	23,382 bp	44,131	Pulcherriminic acid		66%
Region 30.1	NRPS	1 bp	13,447 bp	Fengycin	NRP	20%

When the SZA14 NRP gene cluster (region 6.1; lichenysin) translated sequence was compared to relevant proteins using BLASTp, they showed a significant degree of similarity to proteins involved in the synthesis of variant forms of lichenysin: LicA (100% *B. paralicheniformis*), LiB (100% *B. paralicheniformis*), and LiC (100% *B. licheniformis*). Another NRP cluster (region 30.1; fengycin) displayed 100% identity to non-ribosomal peptide synthetase produced by *B. paralicheniformis*. The translated sequence of region 9.1 NRPs gene cluster was found to be similar to the protein involved in variant forms of bacitracin produced by *B. paralicheniformis*: BacA (99.66%, WP_020452079.1), BacB (100%, AGN36974.1), and BacC (99.98%, WP_154059223.1). The translated sequence of the NRP cluster (bacillibactin; region 1.1) was 99.6% similar to that of non-ribosomal peptide synthetase from *B. subtilis* (WP_025810654). One hybrid gene cluster contained an NRP gene similar to fengycin. The BLASTp-translated sequence showed 99.92% similarity with the non-ribosomal peptide synthetase of *B. subtilis* (WP_059231152.1). Another NRP gene cluster was 99.83% similar to FenA synthetase produced by *B. paralicheniformis*. A Ripp-like gene cluster was found to be 99.12% similar to the uberolysin/carnocyclin family of circular bacteriocin produced by *B. licheniformis* (WP_051303766.1).

The antiSMASH reported gene clusters from the SZA16 genome were similar to the ones detected in the genome of SZA14, implicating their close relatedness. However, when the translated sequence of SZA16 region 2.1 NRP gene cluster was compared to relevant proteins using BLASTp, it showed significant similarity to proteins involved in the synthesis of variant forms of bacitracin produced by *B. paralicheniformis*: BacA (100%, WP_020452079.1), BacB (100%, WP_041817244.1), and BacC (100%, WP_020452077.1). The translated result of another region 4.1 exhibited a high degree of similarity to proteins involved in the synthesis of variant forms of lichenysin: LicA (100%, *B. paralicheniformis*, WP_020450105.1), LiB (100%, *B. paralicheniformis*, WP_020450106.1), and LiC (100%, *B. licheniformis*, AAD04759.1). NRP cluster (region 1.2; bacillibactin) was 100% similar to the non-ribosomal peptide synthetase produced by *B. subtilis* (WP_020453230.1). One hybrid gene cluster (region 3.1; NRPS-betalactone) displayed 100% identity to non-ribosomal peptide synthetase produced by *B. paralicheniformis* (WP_116758584.1). In another cluster (region 14.1, fengycin), the NRP gene cluster displayed 100% identity to non-ribosomal peptide synthetase produced by *B. paralicheniformis* (WP_145697799.1). A Ripp-like gene cluster (region 2.2) was found to be 99.12% similar to the uberolysin/carnocyclin, a family of circular bacteriocins produced by *Bacillus sp.* SB47 (WP_051303766.1).

When the translated sequences of the SZB3 NRP gene clusters (regions 5.1, 6.1, and 11.1) were compared to relevant proteins using BLASTp, they were found to be identical to surfactin non-ribosomal peptide synthetase SrfAC (99.92%, *B. subtilis*; WP_263723575.1), surfactin non-ribosomal peptide synthetase SrfAA (100%, *B. subtilis*; WP_116972609.1), and surfactin non-ribosomal peptide synthetase SrfAB (100%, *B. subtilis*; WP_010886403.1), respectively. A hybrid cluster (region 1.3) showed high similarity to variant forms of plipastatin produced by *B. subtilis*: PpsA (99.92%, WP_009967358.1), PpsB (100%, WP_003247155.1), PpsC (100%, WP_009967356.1), PpsD (100%, WP_009967354.1), and PpsE (99.92%, WP_009967353.1). Another gene cluster (region 2.3; sactipeptide) exhibited 100% identity to subtilosin A, a family of bacteriocins produced by *B. subtilis*. Blastp results for all strains are shown in (Supplementary Table S4).

  BAGEL4 predicted open reading frames (ORFs) coding for ribosomally synthesized proteins and peptides, including bacteriocins and ribosomally synthesized and post-translationally modified peptides (RiPPs). Nine putative gene clusters of interest were reported in the genome of isolate SZA14 that involved genes related to the production of secondary metabolites, including sonorensin, UviB, lasso peptide, bottromycin, enterocin Nkr-5-3B, haloduracin_alpha, comX2, and two sactipeptides. However, due to high relatedness between SZA14 and SZA16 genomes, the six identified gene clusters: enterocin Nkr-5-3B, haloduracin-alpha, sonorensin, bottromycin, comX2, and lasso peptide were similar to the ones observed in the SZA14 genome. Four gene clusters were identified in the genome of isolate_SZB3. Genes for competence, sporulation-killing factor (skfA), subtilosin A (SboA), and sactipeptides were observed (Table 3).

**Table 3:** List of identified RiPPs using BAGEL4 server

Area of interest	Start	End	Class
Predicted RiPPs in SZA14 genome			
lcl_Contig0014.11.AOI_01	1	24121	42.1;haloduracin_alpha
lcl_Contig002.16.AOI_01	585866	606094	272.1;enterocin_Nkr-5-3B
lcl_Contig003.1.AOI_01	34	20382	218.1;sonorensin
lcl_Contig003.1.AOI_02	16367	36367	Bottromycin
lcl_Contig001.20.AOI_01	332234	352234	Lasso peptide
lcl_Contig001.20.AOI_02	211718	231718	Sactipeptide
lcl_Contig0015.2.AOI_01	1313	21313	Sactipeptide
lcl_Contig005.4.AOI_01	76192	96312	319.1;comX2
lcl_Contig0031.7.AOI_01	0	10472	225.2;UviB
Predicted RiPPs in SZA16 genome			
lcl_Contig002.14.AOI_01	212678	232870	272.1;enterocin_Nkr-5-3B
lcl_Contig009.1.AOI_01	1	24412	42.1;haloduracin_alpha
lcl_Contig008.6.AOI_01	112938	133265	218.1;sonorensin
lcl_Contig008.6.AOI_02	129290	149290	Bottromycin
lcl_Contig001.7.AOI_01	975059	995179	319.1;comX2
lcl_Contig001.7.AOI_02	334985	354985	Lasso peptide
Predicted RiPPs in SZB3 genome			
lcl_Contig006.0.AOI_01	160205	180776	121.1;sporulation-killingfactor_skfA
lcl_Contig002.11.AOI_01	378503	399115	216.2;subtilosin_(SboX)
lcl_Contig003.2.AOI_01	67128	87290	492.1;competence
lcl_Contig004.4.AOI_01	67577	87577	Sactipeptides

### Prophage identification and analysis

Full-length genome assemblies for each sample allowed the prediction of the closest putative prophage. A total of 11 prophages were detected in the SZA14 genome using the Phage Hunter server, which included three active and eight ambiguous prophages (Supplementary Table 5a). SZA16 yielded ten prophage candidates, four of which were active and six were ambiguous (Supplementary Table S5b). Whereas, out of the 12 predicted prophage candidates identified in the SZB3 genome, eight were active and four were ambiguous (Supplementary Table S5c).

### Homology modeling

The peptide sequences predicted by the antiSMASH and BAGEL4 servers in the genomes of all three strains were searched for closely related structures on the Phyre2 server. One peptide sequence from SZA14 and SZA16 (referred to as peptide1 hereafter) was found to be closely related to enterocin NKR-5-3B. Whereas, another peptide sequence (referred to as peptide 2) from both SZA14 and SZA16 was closely related to lichenicidin vk21-a1. For SZB3, one peptide sequence was found to be similar to subtilosin A (Table 4).

**Table 4:** Phyre2 query results of peptides

Query peptide	Closely related template	PDB ID	Confidence	Coverage	Identity
SZA14 peptide1	Enterocin NKR-5-3B	2MP8	100.0%	98.0%	58.0%
SZA14 peptide2	Lichenicidin vk21 a1	2KTN	88.2%	49.0%	59.0%
SZA16 peptide1	Enterocin NKR-5-3B	2MP8	100.0%	56.0%	58.0%
SZA16 peptide2	Lichenicidin vk21 a1	2KTN	88.2%	49.0%	59.0%
SZB3 peptide1	Subtilosin A	1PXQ	99.7%	94.0%	97.0%

**Fig. 5:**
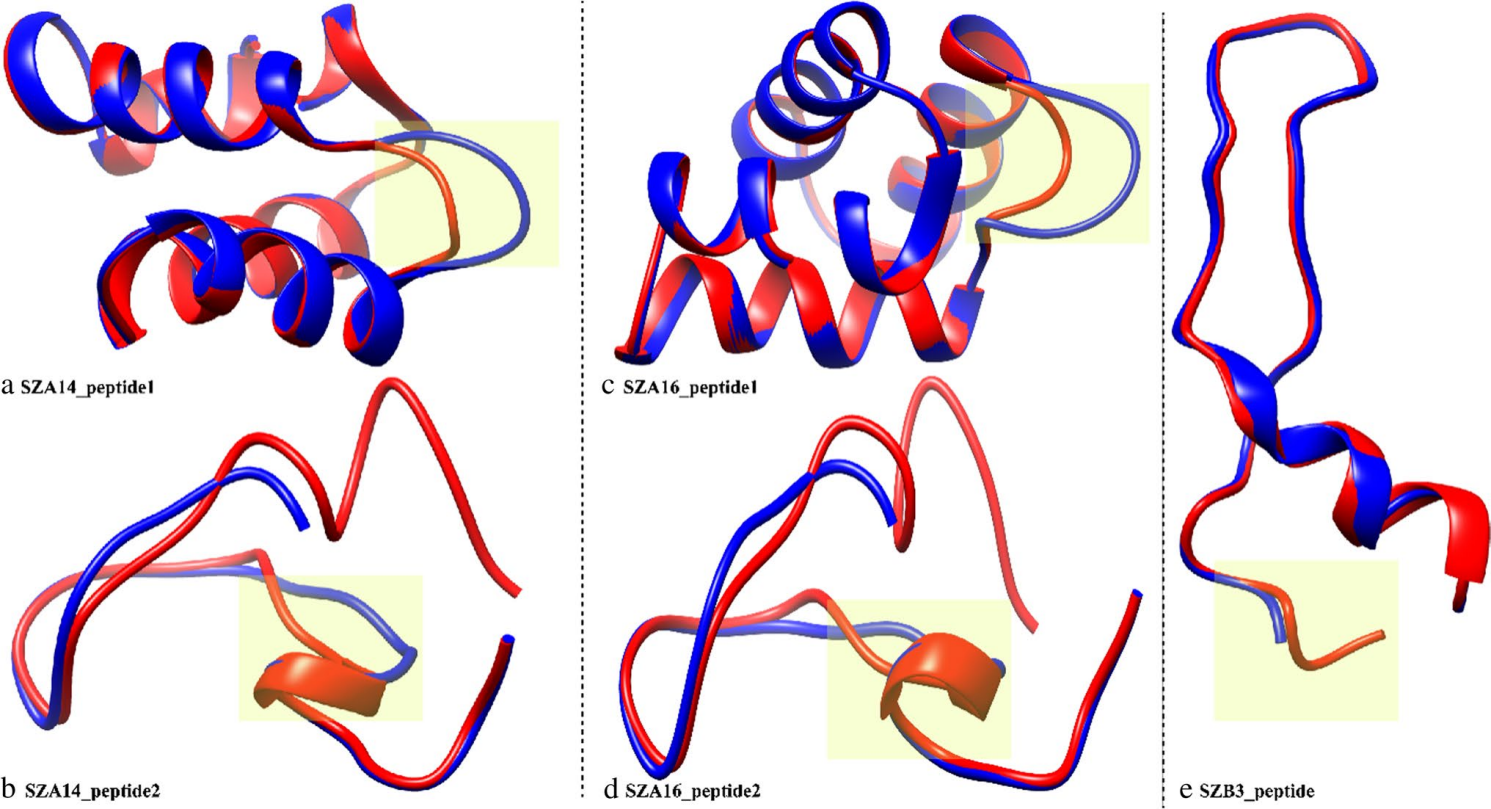
Homology models of the predicted peptide sequences. **a** Superposed structure of SZA14_peptide1 (blue) with enterocin NKR-5-3B PDB ID 2MP8 (red); **b** superposed structure of SZA14_peptide2 (blue) with lichenicidin vk21 a1 PDB ID 2KTN (red); **c** superposed structure of SZA16_peptide1 (blue) with enterocin NKR-5-3B PDB ID 2MP8 (red); **d** superposed structure of SZA16_peptide2 (blue) with lichenicidin vk21 a1 PDB ID 2KTN (red); **e** superposed structure of SZB3_peptide (blue) with subtilosinA PDB ID 1PXQ (red). The highlighted yellow area shows the structural deviation

Homology models of peptides were generated using the Swiss modeling server. Enterocin NKR-5-3B was selected as a template for SZA14_peptide1 (Fig. 5a) and SZA16_peptide1 (Fig. 5c), while lichenicidin vk21-a1 was selected as a template for SZA14_peptide2 (Fig. 5b) and SZA16_peptide2 (Fig. 5d). Subtilosin A was selected as a template for SZB3_peptide1 (Fig. 5e). Peptide structures were superimposed with Chimera, revealing structural deviations at specific positions due to sequence variation as observed by multiple sequence alignment (Supplementary Figure S2). Both SZA14 peptide 1 and SZA16 peptide 1 had a threonine inserted at position 39 in the coil region. Similarly, SZA14_peptide2 and SZA16_peptide2 exhibited deviation at several positions. A seven-residue portion was missing and no modified amino acids were observed. At position 8, a tyrosine was found instead of an alanine. At position 9, asparagine was present in place of isoleucine, and at position 10, the reference leucine was replaced by isoleucine. At position 23, a valine was found in the reference, while peptide 2 had a threonine at that place. At position 26 in peptide 2, leucine insertion was observed. In the SZB3 peptide structure, the first two residues, asparagine and lysine, and modified D-amino acids were missing at positions 28 and 31 compared to the reference subtilosin A sequence.

### Identification of enzymes with biotechnological potential

**Fig. 6:**
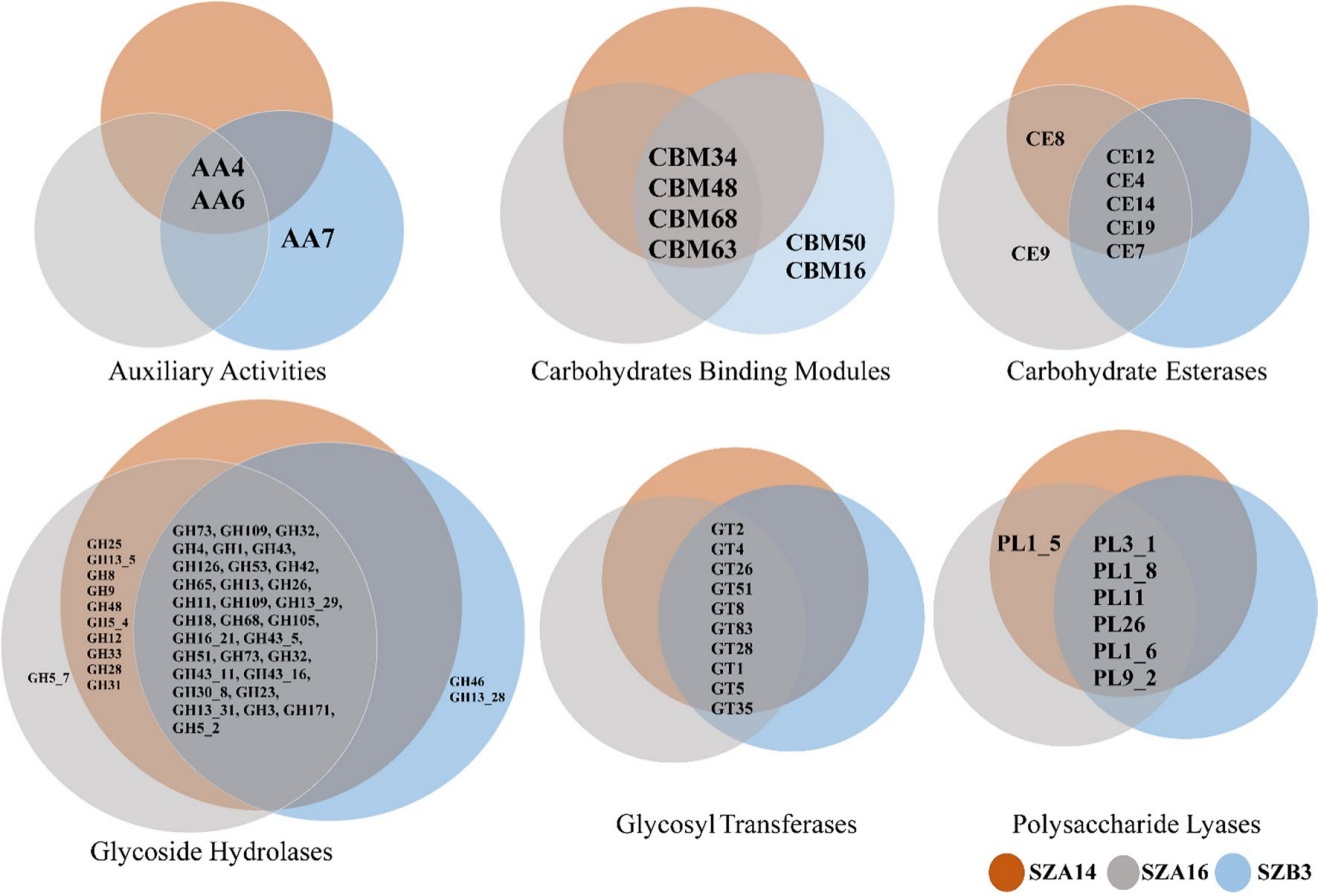
A series of comparative Venn diagrams of the secreted carbohydrate-active enzymes from SZA14 (orange), SZA16 (grey), and the SZB3 strain (blue)

Prokaryotic genome annotation pipeline (PGAP) analysis resulted in 4416, 4270, and 4348 annotated protein sequences for SZA14, SZA16, and SZB3, respectively. The meta server, dbCAN2, classified enzymes and enzymes annotated via all three tools. HMMER: dbCAN, DIAMOND: CAZy, and HMMER: dbCAN-sub were considered significant. From the 4416 genes in the isolate SZA14 genome, 146 were identified by dbCAN as belonging to carbohydrate-active enzyme families. These 146 genes included 40 conserved Carbohydrate-Active enZYme (CAZy) domains whose genes had signal peptides. These CAZy domains represented CAZy families, including two auxiliary activity families (AA), four carbohydrate binding module families (CBM), seven carbohydrate esterase families (CE), 38 glycoside hydrolase families (GH), ten glycosyl transferase families (GT), and seven polysaccharide lyase families (PL). The SZA16 genome encoded 150 CAZy families, with 40 having conserved signal peptides. These CAZy domains include three auxiliary activity families (AA), four carbohydrate binding module families (CBM), seven carbohydrate esterase families (CE), 43 glycoside hydrolase families (GH), ten glycosyl transferase families (GT), and seven polysaccharide lyase families (PL). Alternatively, the SZB3 genome consists of 138 CAZy domains, and 34 of those 138 CAZy domains have conserved signal peptides. Three entries for auxiliary activity families (AA), six carbohydrate binding module families (CBM), seven carbohydrate esterase families (CE), twenty-nine glycoside hydrolase families (GH), ten glycosyl transferase families (GT), and six polysaccharide lyase families (PL). The signal peptide-containing glycoside hydrolase family was the largest group of carbohydrate-active enzymes. A Venn diagram of the annotated enzymes is presented in Fig. 6. EC terms were assigned and the entries were searched manually in the AMFEP list and BRENDA database for their biotechnological potential and industrial applications (Supplementary Table S6).

### Peptides with expected antimicrobial activity

Finally, small peptides in the genomes of SZA14, SZA16, and SZB3 not covered in the previous analyses were identified. The commonly predicted peptides ≤ 100 amino acids by the AMPA (Torrent et al. 2012), ADAM (Lee et al. 2015), and CAMP_R4_ (Waghu et al. 2016) databases were searched against the APD3 database, as shown in Table 5. From the SZA14 genome, eight peptides were predicted. Peptide 2, a 13-residue peptide, exhibited the highest sequence similarity of 53.33% to peptide paenibacterin, followed by peptide 1, a 14-residue peptide showing 42.86% similarity to PE1 and a 13-residue peptide exhibiting 42.86% similarity to polymyxin B. Similarly, from the SZA16 genome, 12 peptides were predicted. Peptide 3, a 13-residue peptide, was found to be similar to paenibacterin with 53.33% similarity while peptide 11, a 12-residue peptide, was 46.15% similar to PE1. Nine peptides were predicted from the SZB3. Peptide 4 was found to be 50% similar to SyCPA-12 and peptide 2 was 43.75% to paenibacterin.

**Table 5:** BLAST results of predicted peptides against APD3 database

S. no	Sequence	APD3 database similarity/APD ID	Peptide name	Organism
SZA14 predicted peptides				
1	K V F I L F Y L W I V P L V	42.86%/AP02201	PE1	*Paenibacillus ehimensis* B7
2	K K V F K S A V K W A P V	53.33%/AP02347	Paenibacterin	*Paenibacillus thiaminolyticus* OSY
3	S K H Q R I K I I K I L E	40%/AP03331	Brevibacillin i	*Brevibacillus laterosporus* DSM 25
4	V K R L T W I I P I V L	40%/AP02243	Gramicidin s	*Bacillus brevis*
5	Y Y R F C H Q N V G R V A R I T	42.11%/AP02376	Lassomycin	*Lentzea kentuckyensis sp*
6	T V A K K R F G L M R I T	42.86%/AP02928	Polymyxin b	*Bacillus aerosporus Greer*
7	L I I K S K W K K V K T	42.86%/AP02204	Colistin a	*Paenibacillus polymyxa* var. colistinus
8	L L I A W R R K R R Q A G	41.18%/AP02678	Plantaricin Y	*Lactiplantibacillus plantarum*
SZA16 predicted peptides				
1	V R L I N K K G T V L I Y R	40%/AP03519	P3	*Brevibacillus sp.* SPR-20
2	L L L I A W R R K R R K A G	41.18%/AP02678	Plantaricin Y	*L. plantarum*
3	K K V F K S A V K W A P V	53.33%/AP02347	Paenibacterin	*Paenibacillus thiaminolyticus* OSY-SE
4	E G K K S K K K N R A F	40%/AP02779	Tridecaptin B1	*Paenibacillus polymyxa* NRRL B-30507
5	N I K L Q R I T L T G H	38.46%/AP02201	PE1	*P. ehimensis* B7
6	V Y N R L L K Y N W F L R K L Y F A L Y	40.91%/AP03562	P4	*Brevibacillus sp.* SPR-20
7	V K R L T W I I P I V L	40%/AP02243	Gramicidin S	*Bacillus brevis*
8	T K K V S G Y I T W Y N G V G K V G	40.91%/AP03460	Triculamin	*Streptomyces triculaminicus* JCM 4242
9	H W H I G Y K F T I F N	38.46%/AP03267	Brevicidine	*B. laterosporus* DSM 25
10	Y E R Y L R L N R R R H K E G G	35.29%/AP02678	Plantaricin Y	*L. plantarum*
11	K R I L K Q K A L F C T	46.15%/AP02201	PE1	*P. ehimensis* B7
12	T V K L V G I Q K R F L P I C	40%/AP02243	Gramicidin S	*B. brevis*
SZB3 predicted peptides				
1	K R V I R Q K A L V C S T	42.86%/AP02201	PE1	*P. ehimensis* B7
2	K T V Y T K Q K M L Q V N K	43.75%/AP02347	Paenibacterin	*P. thiaminolyticus* OSY-SE
3	G I I I Y F I V K Y V M N R N	41.18%/AP03331	brevibacillin I	*B. laterosporus* DSM 25
4	K R L K H A F K P K V T	50%/AP03316	SyCPA 12	*Dickeya dadantii* Ech586
5	T I I H G F K V R P L Y L I	43.75%/AP03326	Bogorol K	*Brevibacillus laterosporus* MG64
6	P K C N A L F L K T K L Q T V	40%/AP02928	Polymyxin B	*B. aerosporus* Greer
7	S Y I V Q T L I V C I A I Y A	42.11%/AP01605	Actagardine	*Actinoplanes garbadinensis* ATCC31048 and 31049
8	V S R L V H L Y G G V I	42.86%/AP02688	Griselimycin	*Streptomyces*
9	R W S K W F N V F C I V A L	41.18%/AP02777	Tridecaptin A1	*Paenibacillus terrae*

## Discussion

For centuries, honey has been used as “folk medicine” (Molan 2015). Honey exhibits an antimicrobial effect against several bacterial (Cooper et al. 2002b, a; Gambo et al. 2018; Grecka et al. 2018) and fungal species (Irish et al. 2006; de Groot et al. 2021). Different factors like the osmotic effect, acidity, hydrogen peroxide, bee defensins, and phytochemicals are attributed to the antimicrobial activity of honey (Brudzynski 2020; Almasaudi 2021; Majtan et al. 2021). Nevertheless, it is also postulated that the additional antimicrobial activity is due to proteinaceous compounds secreted by the gut microbiota of honey bees prior to honey maturation (Mundo et al. 2004; Lee et al. 2008b; Brudzynski 2021). It is now known that raw honey is a reservoir for several microbial species like mold, yeast, and spore-forming bacteria (Snowdon and Cliver 1996).

Recent studies have shown that most of the bacterial isolates recovered from honey can exhibit antimicrobial activity and are a promising source of novel antimicrobials (Lee et al. 2008b, a; Pajor et al. 2018, 2020; Wadi 2022; Xiong et al. 2022). In this study, we isolated three *Bacillus* strains from the honey samples collected from different locations of the same apiary. The isolates exhibited antibacterial activity against the reference *S. aureus* strain both in the overlay inhibition and cell-free supernatant/cell-free after-culture medium (CFAM) assays, following the results obtained by (Pajor et al. 2018).

A large portion of *Bacillus* genome undergoes secondary metabolism and synthesizes diverse secondary metabolites exhibiting broad-spectrum antimicrobial activity (Motta et al. 2008; Li et al. 2012; Sabaté and Audisio 2013). Based on their origin and synthesis pathway, they are classified into two sub-groups: (i) ribosomally synthesized peptides, named bacteriocins, and (ii) non-ribosomally synthesized peptides (NRPs), formed from large enzymatic complexes (Bin Hafeez et al. 2021).

We detected secondary metabolite gene clusters along with lipopeptide antibiotics or proteins in our isolates. The predicted clusters include bacillibactin, butirosin A/B, lichenysin, fengycin, bacitracin, pulcherriminic acid, and amyloliquecidin GF610 from SZA14 and SZA16 and fengycin, bacillaene, thailanstatin A, bacilysin, subtilosin A, bacillibactin, surfactin, pulcherriminic acid, and sporulation-killing factor, from the isolate SZB3.

Bacillibactin (DHB–Gly–Thr) is a cyclic catecholic siderophore (May et al. 2001). Dimopoulou et al. (2021), in a recent study, isolated and characterized the catecholate siderophore bacillibactin from *B. amyloliquefaciens* MBI600 and demonstrated its association with susceptibility in non-susceptible bacterial and fungal phytopathogens. Similarly, Chakraborty et al. (2022) isolated siderophore-type bacillibactin from *Bacillus amyloliquefaciens* MTCC 12,713 and described its bactericidal activity against multidrug-resistant pathogens *S. aureus*, *Klebsiella pneumoniae*, vancomycin-resistant *Enterococcus faecalis*, and *Pseudomonas aeruginosa* causing nosocomial infections (Chakraborty et al. 2022).

Butirosin is an aminoglycosidic antibiotic complex (Dion et al. 1972). It exhibits potent in vitro and in vivo activity against several gram-positive and gram-negative bacteria, including *P. aeruginosa* (Heifetz et al. 1972). However, we observed low sequence identity of butirosin A/B clusters in *B. paralicheniformis*_SZA14 and *B. paralicheniformis*_SZA16 genomes indicating sequence variation and novelty of these honey isolates.

Lichenysin is an anionic lipopeptide produced by *Bacillus* spp (Coronel et al. 2016, 2017). They possess cytotoxic, antimicrobial, and hemolytic activities and have a wide range of potential uses in chemical and biological fields (Ruiz et al. 2017; Coronel et al. 2017). The gene clusters with 100% sequence identity were observed in the genome of *B. paralicheniformis*_SZA14 and *B. paralicheniformis*_SZA16.

Fengycins are cyclic lipo-depsipeptides (CLiPs) synthesized by *Bacillus* spp. and display strong antifungal properties. To date, several variants of fengycin have been described: fengycin A and B, A2, B2, and C2 (Ongena and Jacques 2008), fengycin C (Vater et al. 2002), fengycin D and S (Yang et al. 2015), and fengycin X and Y (Ait Kaki et al. 2020). Apart from antifungal activity, it also exhibits antitumor activity and inhibits human colon cancer HT29 cell line and human lung cancer cell line 95D (Yin et al. 2013; Cheng et al. 2016). The gene clusters observed in the *B. paralicheniformis*_SZA14 and *B. paralicheniformis*_SZA16 genomes displayed a low sequence identity of 20% implicating more in-depth analysis of these regions to look for some novel NRPs.

Bacitracin possesses strong broad-spectrum antibiotic activity against gram-positive bacteria (Weinberg 1967). Luo et al. (2023) reported the production of bacitracin from *B. paralicheniformis* CPL618 and its anti-staphylococcal activity. Bacitracin interferes with cell wall synthesis by inhibiting the dephosphorylation of the lipid carrier (Ming and Epperson 2002). Moreover, it was shown that bacitracin might also cause the degradation of double-stranded DNA (Tay et al. 2010).

Amyloliquecidin is a relatively new two-component (α and β peptides) lantibiotic (Gerst et al. 2022). It is active against most of the tested gram-positive species, which include *Listeria monocytogenes, Clostridium sporogenes, Clostridioides difficile, S. aureus* (Van Staden et al. 2016)*,* and *Alicyclobacillus acidoterrestri* (Gerst et al. 2022)*.* Amyloliquecidin gene clusters within the SZA14 and SZA16 genomes are present in contigs 14 and 9, respectively. While Gerst et al. (2022) in their study reported the presence of amyloliquecidin in contig 19. So far, the gene cluster for lantibiotic amyloliquecidin has been reported in four strains from different locations: *Bacillus* spp*.* 275, isolated in Korea (Gong et al. 2017), *B. amyloliquefaciens* UMAF6639, reported in Spain (Magno-Perez-Bryan et al. 2015), *B. subtilis* subsp. subtilis strain SRCM100333 (Accession: CP021892.1, South Korea), and *B. velezensis* GF610, reported in the USA (Gerst et al. 2022).

Bacillaene is a polyene antibiotic isolated from *B. subtilis* fermentation broths (Mayerl et al. 1995). The compound is produced by the enzymatic megacomplex non-ribosomal peptide synthetases (NRPS) and polyketide synthases (PKS), encoded on a biosynthetic PKS gene cluster (Straight et al. 2007). In agar-plate diffusion experiments, the antibiotic bacillaene is found effective against a wide variety of microorganisms (Mayerl et al. 1995). In vitro studies indicate that bacillaene selectively inhibits prokaryotic protein synthesis (Mayerl et al. 1995). Cell survival studies conducted on *Escherichia coli* indicate its function as a bacteriostatic agent (Mayerl et al. 1995). Bacillaene is thought to play a significant role in *B. subtilis*–based probiotics (Erega et al. 2021; Torres-Sánchez et al. 2021) and functions as an antagonist in a variety of interspecies interactions and prevents *B. subtilis* from predation by *Myxococcus xanthus* (Straight et al. 2007; Yang et al. 2009; Barger et al. 2012).

*B. subtilis*_SZB3 displayed high identity with bacilysin gene cluster, a dipeptide compound produced by spore-forming *Bacillus* causing bacterial and fungal cell lysis (Kenig and Abraham 1976). Nannan et al. (2021) found bacilysin to be a major player in the antagonistic activity of *B. velezensis* against gram-negative foodborne pathogens. The proposed mechanism of action of bacilysin is that it interferes with bacterial cell wall synthesis by inhibiting glucosamine 6-phosphate (GlcN6P) synthase or fungal mannoprotein synthesis (Kenig et al. 1976; Khan et al. 2016). Subtilosin A is a 35-residue macrocyclic peptide encoded by the sbo-albABCDEFG gene cluster (Ishida et al. 2022). The mature peptide is generated from its precursor by proteolytic cleavage of the N-terminal leader peptide and cyclization through covalent bonds between the N-terminal asparagine and the C-terminal glycine (Kawulka et al. 2003). Subtilosin A demonstrates bactericidal activity against *Listeria monocytogenes*, clinical isolates of *Gardnerella vaginalis* and *Streptococcus agalactiae*, and some gram-negative bacteria (Shelburne et al. 2007; Sutyak et al. 2008).

Sporulation-killing factor (SKF) is a ribosomally synthesized and post-translationally modified peptide (Ripp) (González-Pastor et al. 2003). *B. subtilis* exhibits cannibalism under nutrient-stress conditions (González-Pastor et al. 2003). Nandy et al. (2007) in their study reported the Spo0A-mediated killing of *E. coli*, *P. aeruginosa*, *Acinetobactor lwoffi*, and *Xanthomonas campestris* by *B. subtilis* in mixed cultures. Pulcherriminic acid interacts with extracellular ferric ions to form a red pigment pulcherrimin (Kántor et al. 2015). Kántor et al. (2015) reported in their study that pulcherrimin efficiently inhibits the growth of yeast and fungus and also affects the formation of biofilms.

COG analysis revealed S-function unknown to be the dominant COG category, which, however, indicates the potential of these isolates that still needs to be identified. Secondly, the CAZyme analysis provided insight into the enzymatic landscape of the bacteria present in honey. Thiruvengadam et al. (2022) isolated *B. subtilis* Bbv57 from the betel vine rhizosphere and found glycosyl transferases and glycoside hydrolases present predominantly in the genome. Endo β 1, 4 glucanase, chitinase, endoglucanase, and xyloglucanase were also detected in their study (Thiruvengadam et al. 2022). In another study, Chen et al. (2022) isolated *B. subtilis* CTXW 7–6-2 from kiwi fruit and observed the same results. These enzymes, such as glycosyl transferases, glycoside hydrolases, and carbohydrate esterase-related enzymes, are considered to be involved in the synthesis of secondary metabolites via non-ribosomal pathways, while others like chitinase, chitosanase, endoglucanase, and lysozyme can possibly be involved in the synthesis of antifungal CAZymes (Kumar et al. 2019; Chen et al. 2022). A similar CAZymes landscape observed in our isolates draws attention towards the secretion of these enzymes/products into the medium (honey), enhancing their therapeutic potential.

The hypothetical proteins and peptides were analyzed using AMPA, ADAM, and CAMPR4 algorithms to predict their antimicrobial potential (Pavlova et al. 2020). However, given the low accuracy and prediction power of peptide tools, experimental determination of peptides and their activities is necessarily required for further research into these potential AMPs. Structural variations observed by multiple sequence alignments and homology modeling enabled us to observe the missing residues observed in the structure of the peptides as a result of the directly translated gene product, which would otherwise have undergone extensive post-translational modifications by bacterial machinery inside a cell. However, multiple sequence alignments still depict the expected changes in the peptides.

Phage-mediated recombination is an essential event for environmental bacteria to exchange genetic information, which confers a variety of favorable traits like evolution and adaptability as well as the acquisition of antibiotic resistance or producing genes (Iqbal et al. 2023). We reported the presence of prophages in the genomes of all three isolates: SZA14, SZA16, and SZB3. We hypothesize that active prophages might be responsible for some important enzyme synthesis and stress-related proteins and even antimicrobial peptides. However, no detailed analysis was performed regarding phage hunting.

In this study, we reported three bacterial isolates able to produce potent antimicrobial peptides and enzymes with biotechnological potential. In addition, we also found the predominant presence of *Bacillus* sp. in the majority of the samples. However, extensive wet lab experiments assisted by computational studies are needed to further exploit the potential of these isolates.

### Supplementary Information

Below is the link to the electronic supplementary material.

Supplementary file1 (PDF 1836 KB)

## Data Availability

The data presented in this study are available on request from the corresponding author.
