# Supplementary material for: Whole-genome sequencing and antimicrobial potential of bacteria isolated from Polish honey
Source: Appl Microbiol Biotechnol. 2023 Sep 4;107(20):6389–406. doi: 10.1007/s00253-023-12732-9 (PMC10560198; doi:10.1007/s00253-023-12732-9)
Supplement: Supplementary file 1 — Supplementary file1 (PDF 1836 KB) [file 253_2023_12732_MOESM1_ESM.pdf]

Springer

Applied Microbiology and Biotechnology

Whole-genome sequencing and antimicrobial potential of bacteria isolated from Polish honey

Ahmer Bin Hafeez[1]; Karolina Pełka[1]; Kamila Buzun[2]; Randy Worobo[3]; Piotr Szweda[1]*

1 - Department of Pharmaceutical Technology and Biochemistry, Faculty of Chemistry, Gdańsk University of

Technology, ul. G. Narutowicza 11/12, 80-233 Gdańsk, Poland;

2 – Department of Biotechnology, Faculty of Pharmacy, Medical University of Bialystok, 15-089 Bialystok, Poland

3 - Department of Food Science, Cornell University, Ithaca, NY 14853, USA

Ahmer Bin Hafeez – e-mail: ahmer.bin.hafeez@pg.edu.pl; ORCID: 0000-0002-8712-5898

Karolina Pełka – e-mail: karolina.pelka@pg.edu.pl; ORCID: 0000-0003-2523-8165

Kamila Buzun – e-mail: buzunkamila@gmail.com; ORCID: 0000-0002-5030-2862

Randy Worobo – e-mail: rww8@cornell.edu; ORCID: 0000-0002-5211-3125

Piotr Szweda – email: piotr.szweda@pg.edu.pl; ORCID: 0000-0001-8291-5148*

Table S1. List of publicly available *Bacillus* spp. genome assembly included in this study.

Isolate	Assembly Accession Number
*B. paralicheniformis*_Strain_NCTC 8721	GCA_007832015.1
*B. paralicheniformis*_Strain_CBMAI1303	GCA_003711025.1
*B. paralicheniformis*_Strain_TXO7B-1SG6	GCA_019931655.3
*B. paralicheniformis*_Strain_ATCC9945a	GCA_000408885.1
*B. paralicheniformis*_Strain_FA6	GCA_009497935.1
*B. paralicheniformis*_Strain_HAS-1	GCA_019931595.1
*B. paralicheniformis*_Strain_J36TS2	GCA_021654735.1
*B. subtilis*_DSM10	GCA_014389245.2
*B. subtilis*_strain_P9_B1	GCA_009662455.1
*B. subtilis*_strain_168	GCA_013009385.1
*B. subtilis*_strain_Bsi	GCA_021398545.1
*B. subtilis*_strain_FJAT-4	GCA_019931715.1
*B. subtilis*_strain_KCTC3135	GCA_001697265.1
*B. cappridis*_strain_DSM 103394	GCA_017832095.1

Table S2. QUAST report summary

**QUAST summary on_SZA14_Assembly**	
# contigs (>= 0 bp)	54
# contigs (>= 1000 bp)	40
Total length (>= 0 bp)	4509931
Total length (>= 1000 bp)	4502525
# contigs	46
Largest contig	675381
Total length	4507369
GC (%)	45.59
N50	275618
N75	128457
L50	5
L75	11
# N's per 100 kbp	0.00
**QUAST summary on_SZA16_Assembly**	
# contigs (>= 0 bp)	29
# contigs (>= 1000 bp)	19
Total length (>= 0 bp)	4381813
Total length (>= 1000 bp)	4377371
# contigs	21
Largest contig	1077224
Total length	4377317
GC (%)	45.81
N50	668104
N75	316902
L50	3
L75	5
# N's per 100 kbp	0.00
**QUAST summary on_SZB3_Assembly**	
# contigs (>= 0 bp)	25
# contigs (>= 1000 bp)	14
Total length (>= 0 bp)	4234695
Total length (>= 1000 bp)	4229692
# contigs	19
Largest contig	2254600
Total length	4232608
GC (%)	43.40
N50	2254600
N75	487702

L50	1
L75	3
# N's per 100 kbp	0.00

Table S3a. List of identified Secondary metabolite cluster of SZA16 using strictness 'strict'

Region	Type	From	To	Most similar known cluster		Similarity
Region 1.1	lassopeptide	334,914	357,375			
Region 1.2	NRP-metallophore, NRPS	460,403	507,569	bacillibactin/bacillibactin E/bacillibactin F	NRP	100%
Region 1.3	CDPS	766,360	787,109	pulcherriminic acid		66%
Region 2.1	NRPS	40,553	123,442	bacitracin	NRP	100%
Region 2.2	RiPP-like	217,676	228,020			
Region 3.1	NRPS, betalactone	629,597	668,104	fengycin	NRP	60%
Region 4.1	NRPS	190,996	256,433	lichenysin	NRP	100%
Region 6.1	T3PKS	64,076	105,173			
Region 6.2	terpene	158,320	180,209			
Region 6.3	NRPS	275,513	297,297	fengycin	NRP	20%
Region 7.1	NRPS-independentsiderophore	190,208	205,671	schizokinen		60%
Region 8.1	thiopeptide, RiPP-like	109,022	150,264	butirosin A / butirosin B	Saccharide	7%
Region 9.1	lanthipeptideclass-ii, cyclic-lactoneautoinducer	1	25,446	amyloliquecidin GF610	RiPP	93%
Region 14.1	NRPS	1	13,447	fengycin	NRP	20%

Table S3b. List of identified Secondary metabolite cluster of SZB3 using strictness 'strict'

Region	Type	From	To	Most similar known cluster		Similarity
Region 1.1	T3PKS	877,830	918,720	1-carbapen-2-em-3-carboxylic acid		16%
Region 1.2	terpene	967,671	988,526			
Region 1.3	NRPS, betalactone	1,063,765	1,140,800	fengycin	NRP	100%
Region 1.4	NRPS, transAT-PKS, T3PKS	1,337,705	1,442,951	bacillaene	polyketide+NRPS	100%
Region 1.5	terpene	2,035,973	2,056,776			
Region 2.1	Epipeptide	87,878	109,576	thailanstatin A	NRP + polyketide	100%
Region 2.2	other	333,112	374,530	bacilysin	Other	100%
Region 2.3	sactipeptide	377,529	399,140	subtilosin A	RiPP:thiopeptide	100%
Region 3.1	NRP-metallophore, NRPS	81,790	128,926	bacillibactin	NRP	100%
Region 3.2	CDPS	415,093	435,839	pulcherriminic acid	Other	100%
Region 5.1	NRPS	1	25,185	surfactin	NRP:lipopeptide	43%
Region 6.1	NRPS	1	27,752	surfactin	NRP:lipopeptide	43%
Region 6.2	sactipeptide, ranthipeptide	157,825	180,778	sporulation killing factor	RiPP:head-to-tailcyclized peptide	100%
Region 11.1	NRPS	1	9,073	surfactin	NRP:lipopeptide	8%

Table S4a. BLASTp results of SZA14 gene clusters

Region	Description	Scientific Name	Max Score	Total Score	Query Cover	E value	Per. Ident	Acc. Len	Accession
Region 1.1	non-ribosomal peptide synthetase	*B. subtilis* group	4887	4887	100%	0.0	99.96%	2388	WP_025810654.1
Region 1.2	lasso peptide biosynthesis B2 protein	*Bacillus sp.* SB47	318	318	100%	1e-109	99.35%	154	WP_026580276.1
Region 2.1	uberolysin/carnocyclin family circular bacteriocin	*B. licheniformis*	217	217	100%	1e-^{70}	99.12%	114	WP_051303766.1
Region 3.1	putative thiazolecontaining bacteriocin maturation protein	*B. paralicheniformis*	1268	1268	100%	0.0	99.68%	618	WP_216904229.1
Region 3.2	IucA/IucC family siderophore biosynthesis protein	*B. paralicheniformis*	1209	1209	100%	0.0	99.83%	587	WP_145685709.1
Region 6.1	lichenysin nonribosomal peptide synthetase LicA/B/C*	*B. paralicheniformis/Licheniformis**	7380 7406 2659	7380 7406 2659	100% 100% 100%	0.0 0.0 0.0	100.00 % 100.00 %100.00%	3582 3589 1288	WP_075213809.1 WP_075213808.1 AAD04759.1*
Region 7.1	non-ribosomal peptide synthetase	*B. subtilis* group	2629	2629	100%	0.0	99.92%	1267	WP_059231152.1
Region 8.1	FenA	*B. paralicheniformis*	1239	1435	100%	0.0	99.83%	1708	VEB19239.1
Region 8.2	sporulenol synthase	*B. paralicheniformis*	1310	1310	100%	0.0	100.00 %	674	TWN88759.1
Region 9.1	bacitracin nonribosomal peptide synthetase BacA/B/C	*B. paralicheniformis*	10839 5399 13143	10839 5399 13143	100% 100% 100%	0.0 0.0 0.0	99.66% 100.00 % 99.98%	5256 2607 6359	WP_020452079.1 AGN36974.1 WP_154059223.1
Region 11.1	chalcone synthase	*B. paralicheniformis*	751	751	100%	0.0	100.00 %	365	WP_025811248.1
Region 14.1	type 2 lanthipeptide synthetase LanM	*B. subtilis* group	2287	2287	100%	0.0	99.91%	1105	WP_223254889.1

Region 23.1	tRNA-dependent cyclodipeptide synthase	*Bacillus sp.* B19-2	513	513	100%	0.0	99.60%	249	WP_059231995.1
Region 30.1	non-ribosomal peptide synthetase	*B. paralichenifo rmis*	2709	2709	100%	0.0	100.00 %	1388	WP_145697799.1

Table S4b. BLASTp results of SZA16 gene clusters

**Region**	**Description**	**Scientific Name**	**Max Score**	**Total Score**	**Query Cover**	**E value**	**Per. Ident**	**Acc. Len**	**Accession**
Region 1. 1	lasso peptide biosynthesis B2 protein	*B. subtilis* group	322	322	100%	6e-111	100.00%	154	WP_020453359.1
Region 1. 2	non-ribosomal peptide synthetase	*B. subtilis* group	4881	4881	100%	0.0	100.00%	2388	WP_020453230.1
Region 1. 3	tRNA-dependent cyclodipeptide synthase	*B. subtilis* group	514	514	100%	0.0	100.00%	249	WP_020452964.1
Region 2. 1	bacitracin nonribosomal peptide synthetase BacA/BacB/Bac C	*B. paralicheniformis*	1086<br>7540<br>0<br>1314<br>8	10867<br><br>5400<br>13148	100%<br>100%<br>100%	0.0<br>0.0<br>0.0	100.00%<br>100.00%<br><br>100.00%	5256<br>2605<br>6359	WP_020452079.1<br>WP_041817244.1<br>WP_020452077.1
Region 2. 2	uberolysin/carnoc yclin family circular bacteriocin	*Bacillus sp*. SB47	217	217	100%	9e-71	99.12%	114	WP_051303766.1
Region 3. 1	non-ribosomal peptide synthetase	*B. paralicheniformis*	2683	2683	100%	0.0	100.00%	1290	WP_116758584.1
Region 4. 1	lichenysin nonribosomal peptide synthetase LicA/LicB/LicC	*B. paralicheniformis/l icheniformis* (LicC)	7388<br>7395<br>2659	7388<br>7395<br><br>2659	100%<br>100%<br>100%	0.0<br>0.0<br><br>0.0	100.00%<br>100.00%<br>100.00%	3582<br>3587<br>1288	WP_020450105.1<br>WP_020450106.1<br>AAD04759.1
Region 6.1	chalcone synthase BcsA	*B. subtilis* group	753	753	100%	0.0	100.00%	365	WP_020451900.1
Region 6.2	sporulenol synthase	*B. paralicheniformis*	1310	1310	100%	0.0	100.00%	674	TWL48322.1
Region 6.3	FenA	*B. paralicheniformis*	1239	1435	100%	0.0	99.83%	1708	VEB19239.1

Region 7.1	rhizobactin siderophore biosynthesis protein RhbC	*B. subtilis* group	1211	1211	100%	0.0	100.00%	587	WP_020450899.1
Region 8.1	putative thiazolecontaining bacteriocin maturation protein	*B. subtilis* group	1267	1267	100%	0.0	100.00%	617	WP_020450760.1
Region 9.1	type 2 lanthipeptide synthetase LanM	*B. subtilis* group	2287	2287	100%	0.0	99.91%	1105	WP_223254889.1
Region 14.1	non-ribosomal peptide synthetase	*B. paralicheniformis*	2709	2709	100%	0.0	100.00%	1388	WP_145697799.1

Table S4c. BLASTp results of SZB3 gene clusters

Region	Description	Scientific Name	Max Score	Total Score	Query Cover	E value	Per. Ident	Acc. Len	Accession
Region 1.1	terpene family molecule synthase	*B. subtilis* QB928	755	755	100%	0.0	100%	400	AFQ58153.1
Region 1.2	squalene-hopene cyclase	*B. subtilis* QB928	1325	1325	100%	0.0	100.00%	640	AFQ57872.1
Region 1.3	non-ribosomal plipastatin synthetase PpsA/PpsB/PpsC /PpsD/PpsE	*B. subtilis*	5303<br>5325<br>5291<br>7487<br>2655	5303<br>5325<br>5291<br>7487<br>2655	100%1<br>00%10<br>0%100<br>%100<br>%	0.0 0.0<br>0.0<br>0.0<br>0.0	99.92%<br>100.00%<br>100.00%<br>100.00%<br>99.92%	2561<br>2560<br>2555<br>3603<br>1272	WP_009967358.1<br>WP_003247155.1<br>WP_009967356.1<br>WP_009967354.1<br>WP_009967353.1
Region 1.4	polyketide synthase PksG/PksJ/PksL/ PksM/PksN	*B. subtilis*	875<br>10495<br>9424<br>8883<br>11457	875<br>1049<br>5<br>9424<br>8883<br>1145<br>7	100%<br>100%1<br>00%10<br>0%<br>100%	0.0<br>0.0<br>0.0<br>0.0<br>0.0	100.00%<br>100.00%<br>99.98%<br>100.00%<br>99.98%	420<br>5043<br>4532<br>4262<br>5514	WP_003231805.1<br>WP_003245563.1<br>AHA77747.1<br>WP_003245093.1<br>BBK72416.1
Region 1.5	farnesyl diphosphate phosphatase	*B. subtilis*	553	553	100%	0.0	100.00%	267	WP_003245866.1
Region 2.1	YydG family radical SAM peptide epimerase	*B. subtilis*	656	656	100%	0.0	99.69%	319	WP_159377167.1
Region 2.2	alanineanticapsin ligase	*B. subtilis*	972	972	100%	0.0	99.79%	472	WP_217002782.1

Region 2.3	subtilosin A family bacteriocin	*B. subtilis*	83.6	83.6	95%	7e-20	100.00%	41	WP_212036318.1
Region 3.1	DhbF	*B. subtilis*	4857	4857	100%	0.0	99.96%	2378	AAD56240.1
Region 3.2	tRNA-dependent cyclodipeptide synthase	*B. subtilis*	508	508	100%	0.0	99.60%	248	WP_213397052.1|
Region 5.1	surfactin nonribosomal peptide synthetase SrfAC	*B. subtilis*	2645	2645	100%	0.0	99.92%	1275	WP_263723575.1
Region 6.1	surfactin nonribosomal peptide synthetase SrfAA	*B. subtilis*	5335	6382	100%	0.0	100.00%	2626	WP_116972609.1
Region 6.2	sporulation killing factor system radical SAM maturase/sporulation killing factor biosynthesis protein SkfC/ sporulation killing factor*	*B. subtilis/ Bacillus atrophaeus**	853<br>1005<br>111*	853<br>1005<br>111*	100%<br>100%<br>100%*	0.0<br>0.0<br>2e-30*	99.76%<br>99.80%<br>98.18%*	410<br>496<br>55*	WP_128992337.1<br>WP_128992338.1<br>WP_035174941.1*
Region 11.1	surfactin nonribosomal peptide synthetase SrfAB	*B. subtilis*	5326	7277	100%	0.0	100.00%	3583	WP_010886403.1

Table S5a. List of proposed prophages predicted by Phage hunter in the SZA14 genome

Candidate ID	Sequence ID	Start	End	Length	Category	Score	Closest phage	Gene number
Candidate_19	lcl|Contig001	753269	767653	14385	Ambiguous	0.74	*Staphylococcus* phage StauST398-5	14
Candidate_27	lcl|Contig001	1041726	1062773	21048	Active	0.84	*Enterococcus* phage EFDG1	24
Candidate_48	lcl|Contig002	485100	553385	68286	Active	0.86	*Bacillus* phage phi105	84
Candidate_49	lcl|Contig002	530477	547321	16845	Active	0.83	*Bacillus* phage phi105	20
Candidate_53	lcl|Contig002	704982	726134	21153	Ambiguous	0.53	*Pectobacterium* phage CBB	21
Candidate_62	lcl|Contig003	313117	337866	24750	Ambiguous	0.72	*Escherichia* phage vB_EcoM_VR25	25
Candidate_67	lcl|Contig003	524128	546304	22177	Ambiguous	0.53	*Bacillus* phage phi3T	32
Candidate_84	lcl|Contig005	84700	120944	36245	Ambiguous	0.53	*Flavobacterium* phage FL-1	30
Candidate_88	lcl|Contig006	1125	25068	23944	Ambiguous	0.56	*Bacillus* phage 0305phi8-36	26
Candidate_90	lcl|Contig006	54037	77833	23797	Ambiguous	0.60	N/A	27
Candidate_99	lcl|Contig007	29706	79151	49446	Ambiguous	0.62	*Bacillus* phage vB_BtS_BMBtp14	59

Table S5b. List of proposed prophages predicted by Phage hunter in the SZA16 genome

Candidate ID	Sequence ID	Start	End	Length	Category	Score	Closest phage	Gene number
Candidate_26	NODE_1_length _1077535	753412	767796	14385	Ambiguous	0.74	*Staphylococcus* phage StauST398-5	14

Candidate ID	Sequence ID	Start	End	Length	Category	Score	Closest phage	Gene number
Candidate_34	NODE_1_length_1077535	1041869	1062916	21048	Active	0.84	*Enterococcus* phage EFDG1	24
Candidate_49	NODE_2_length_826361	485227	553512	68286	Active	0.86	*Bacillus* phage phi105	84
Candidate_50	NODE_2_length_826361	530604	547448	16845	Active	0.83	*Bacillus* phage phi105	20
Candidate_54	NODE_2_length_826361	705109	726261	21153	Ambiguous	0.53	*Pectobacterium* phage CBB	21
Candidate_63	NODE_3_length_668380	313266	338015	24750	Ambiguous	0.72	*Escherichia* phage vB_EcoM_VR25	25
Candidate_68	NODE_3_length_668380	524277	546453	22177	Ambiguous	0.53	*Bacillus* phage phi3T	32
Candidate_85	NODE_5_length_297551	54164	77960	23797	Ambiguous	0.60	N/A	27
Candidate_94	NODE_6_length_284625	1868	54432	52565	Active	0.88	*Bacillus* phage phi105	68
Candidate_102	NODE_7_length_242040	26253	81427	55175	Ambiguous	0.52	*Bacillus* phage vB_BtS_BMBtp14	65

Table S5c. List of proposed prophages predicted by Phage hunter in the SZB3 genome

Candidate ID	Sequence ID	Start	End	Length	Category	Score	Closest phage	Gene number

Candidate ID	Sequence ID	Start	End	Length	Category	Score	Closest phage	Gene number
Candidate_7	lcl|Contig001	308229	350203	41975	Active	0.93	*Bacillus* phage phi105	53
Candidate_10	lcl|Contig001	499885	566461	66577	Ambiguous	0.68	*Bacillus* phage phi3T	87
Candidate_11	lcl|Contig001	551100	566833	15734	Ambiguous	0.55	*Bacillus* phage phi3T	18
Candidate_27	lcl|Contig001	1197264	1271138	73875	Ambiguous	0.69	*Bacillus* phage phi3T	106
Candidate_28	lcl|Contig001	1271540	1302294	30755	Active	0.97	*Bacillus* phage phi3T	38
Candidate_29	lcl|Contig001	1281182	1347517	66336	Ambiguous	0.68	*Bacillus* phage phi3T	73
Candidate_38	lcl|Contig001	1849522	1892675	43154	Active	0.86	*Bacillus* phage phi105	52
Candidate_55	lcl|Contig002	195824	209295	13472	Active	0.84	N/A	10
Candidate_73	lcl|Contig003	443527	467144	23618	Active	0.92	N/A	27
Candidate_92	lcl|Contig007	36067	56966	20900	Active	0.92	*Listeria* phage vB_LmoS_188	35
Candidate_96	lcl|Contig009	304	20295	19992	Active	0.98	*Brochothrix* phage NF5	35
Candidate_97	lcl|Contig009	210	52888	52679	Active	0.93	*Bacillus* phage phi3T	76

Table S6a. Potential biotechnologically important enzymes annotated from strain SZA14 strain

Enzyme Name	EC	Length	Identity (%)	Producer Organism	Accession number	Applications
Pectate lyase	4.2.2.2	221	96.8	*B. amyloliquefaciens/subtilis*	Q65EF5	Agriculture, Biotechnology, degradation, environmental protection, food industry, industry & molecular biology
N-acetylglucosaminidase (Major autolysin)	3.2.1.17	877	44.6	*Streptomyces violaceoruber, B. licheniformis*	Q65E77	Agriculture, enzymatic activity analysis, food industry, industry, medicine & synthesis
β-fructofuranosidase (inveratse)	3.2.1.26	478	94.1	*Saccharomyces cerevisiae*	Q65DN5	Agriculture, enzymatic activity analysis, biofuel production, food industry, industry, pharmacology & synthesis
Pectate disaccharide-lyase	4.2.2.9	341	97.7	*Acrophialophora nainiana*	B1B6T1	Industry
β-galactosidase	3.2.1.23	472	97.5	*Aspergillus oryzae, Bacillus circulans, Kluyveromyces lactis*	Q65D52	enzymatic activity analysis, biofuel production, Biotechnology, degradation, diagnostic, food industry, industry, medicine, molecular biology & synthesis
β-glucosidase	3.2.1.21	469	97.2	*Aspergillus niger, Penicillium decumbens, Penicillium multicolor, Trichoderma sp.*	Q65D37	enzymatic activity analysis, biofuel production, Biotechnology, degradation, food industry, industry, medicine, molecular biology & synthesis
Maltogenic amylase	3.2.1.133	588	77.1	*B. amyloliquefaciens/subtilis/ licheniformis/Bacillus stearothermophilus*	O06988	Biotechnology, degradation, food industry, medicine, pharmacology & synthesis
α-glucosidase	3.2.1.20	561	84.7	*Trichoderma reesei or longibrachiatum, A. niger*	O06994	Agriculture, Biotechnology, diagnostic, medicine, nutrition, synthesis
Mannan endo-1,4β-mannosidase	3.2.1.78	360	95.3	*A. niger, Trichoderma sp.*	Q65MP4	Agriculture, Biotechnology, degradation, drug diagnostic, food industry, industry, paper production, pharmacology & synthesis
Penicillin-binding protein	2.4.1.129	895	65.9	*B. subtilis*	P39793	Drug development, medicine
Endo-1,4-βxylanase	3.2.1.8	213	91.5	*A. niger/oryzae, B. amyloliquefaciens/subtilis/ licheniformis, Disporotrichum dimorphosporum, Humicola insolens, Penicillium or Talaromyces emersonii, Streptomyces violaceoruber, Trichoderma sp.*	P18429	Agriculture, analysis, , biofuel production, Biotechnology, degradation, food industry, industry, paper production, paper production & synthesis
Pullulanase	3.2.1.41	718	55.6	*Bacillus acidopullulyticus/licheniformis, amyloliquefaciens/subtilis,*	C0SPA0	Biotechnology, degradation, food industry, synthesis

				Pullulanibacillus naganoensis		
Branching glycosyltransferase	2.4.1.18	627	96.2	*B. amyloliquefaciens/subtilis, Geobacillus stearothermophilus*	Q65FS4	Biotechnology, Drug development, food industry, medicine, nutrition
Glucanase (endo1,3(4)-β)	3.2.1.73	242	90.9	*A. niger/oryzae, B. amyloliquefaciens/subtilis, Cellulosimicrobium cellulans, Disporotrichum dimorphosporum, Humicola insolens, Talaromyces emersonii, Trichoderma harzianum/reesei/longibrachiatum*	P04957	Agriculture, enzymatic activity analysis, biotechnology, degradation, drug development, food industry, industry & synthesis
Arabinofuranosidase	3.2.1.55	502	75.9	*A. niger*	Q9XBQ3	Agriculture, enzymatic activity analysis, biofuel production, Biotechnology, degradation, drug development, food industry, industry, medicine, nutrition, paper production & synthesis
β-fructofuranosidase	3.2.1.80	677	79	*B. subtilis, S. cerevisiae*	P05656	Agriculture, enzymatic activity analysis, biofuel production, food industry, industry, Pharmacology & synthesis
Xylan 1,4-βxylosidase	3.2.1.37	533	83.6	*A. niger, B. subtilis/pumilus*	P94489	Agriculture, enzymatic activity analysis, biofuel production, biotechnology, degradation, food industry, industry, nutrition, pharmacology & synthesis
Amylase (α)	3.2.1.1	512	96.5	*A. niger/oryzae, B. amyloliquefaciens/subtilis/licheniformis/stearothermophilus, Microbacterium imperial, Streptomyces violaceoruber, T. reesei/longibrachiatum*	P06278	Agriculture, enzymatic activity analysis, biofuel, brewing, detergent, energy production, biotechnology, food industry, industry, medicine, pharmacology & synthesis
α-galactosidase	3.2.1.22	432	9.9	*A. niger, S. cerevisiae*	O34645	enzymatic activity analysis, Biotechnology, degradation, medicine, molecular biology, nutrition & synthesis
Cellulase	3.2.1.4	560	97	*A. niger B. amyloliquefaciens/subtilis, Penicillium funiculosum, Talaromyces emersonii, Streptomyces lividans, T. reesei/longibrachiatum*	Q65JI7	Agriculture, enzymatic activity analysis, biofuel, degradation, detergent, energy production, biotechnology, diagnostic, environmental protection, food industry, industry, textile, & synthesis
Pectinesterase	3.1.1.11	317	9702	*A. niger/oryzae, B. licheniformis*	Q65F39	Diagnostic, food industry, industry
Endo-polygalacturonase	3.2.1.15	453	60.5	*A. niger, T. reesei/longibrachiatum*	A0A075K9K3	Agriculture, enzymatic activity analysis, degradation, food industry, industry, medicine, nutrition and paper production
Chitinase	3.2.1.14	598	64.6	*Streptomyces violaceoruber, Bacillus clausii*	Q5WKC0	Agriculture, enzymatic activity analysis, degradation, , biotechnology, environmental

						protection, industry, medicine, molecular biology, pharmacology & synthesis

Table S6b. Potential biotechnologically important enzymes annotated from strain SZA16 strain

Enzyme Name	EC	Length	Identity (%)	Producer Organism	Accession number	Applications
Glycogen phosphorylase	2.4.1.1	798	76.9	*B. subtilis/licheniformis/gobiensis/indicus*	P39123	Agriculture, enzymatic activity analysis, Drug development, Biotechnology, medicine & synthesis
Levansucrase	2.4.1.10	473	76.8	*B. subtilis/licheniformis/amyloliquefaciens, Zymomonas mobilis*	P05655	Agriculture, Food industry, synthesis
Starch [bacterial glycogen] synthase	2.4.1.21	484	91	*Arabidopsis thaliana, Oryza sativa, Triticum aestivum,*	Q65FS7	Agriculture, Biofuel, Food industry, nutrition
Cephalosporin-C deacetylase	3.1.1.41	318	76.6	*B. subtilis/ licheniformis/pumilus, Brevibacillus fluminis*	P94388	synthesis
β-N-acetylhexosaminidase	3.2.1.52	642	67.8	*Aspergillus niger/oryzae, B. subtilis, Fusarium oxysporum, Kluyveromyces lactis*	P40406	enzymatic activity analysis, Drug development, diagnostic, food industry & medicine
Endo-1,3(4)-βglucanase	3.2.1.6	242	90.5	*A. niger/oryzae, B. amyloliquefaciens/subtilis, Cellulosimicrobium cellulans, Disporotrichum dimorphosporum*	P04957	Agriculture, enzymatic activity analysis, Drug development, Biotechnology, food industry, nutrition & synthesis
Extracellular endoα-(1->5)-Larabinanase 2	3.2.1.99	469	75.6	*B. subtili s/licheniformis, A. niger, Caldicellulosiruptor saccharolyticus, Geobacillus stearothermophilus*	P42293	Enzymatic activity analysis, Biofuel, degradation, industry, nutrition & synthesis
Peptidoglycan-Nacetylglucosamine deacetylase	3.5.1.104	263	76.9	*B. subtilis/licheniformis/pumilus, Lactococcus lactis, Streptococcus pneumoniae*	O34928	Medicine, pharmacology
N-acetylglucosamine6-phosphate deacetylase	3.5.1.25	395	59.7	*Bacillus sp. V59.32b, Streptococcus pneumonia, Mycolicibacterium smegmatis, Komagataeibacter xylinus*	A0A3E2JBY3	Diagnostics, Drug development

Table S6c. Potential biotechnologically important enzymes annotated from strain SZB3 strain

**Enzyme Name**	**EC**	**Length**	**Identity (%)**	**Producer Organism**	**Accession number**	**Applications**

Glycogen phosphorylase	2.4.1.1	256	100	*B.subtilis/pumilus/licheniformis, Aquifex aeolicus, Azospirillum brasilense, Artemia sinica*	P27620	Agriculture, Enzymatic activity analysis, Biotechnology, drug development, medicine and synthesis
Levansucrase	2.4.1.10	473	100	*Lolium perenne, B.subtilis /licheniformis/amyloliquefaciens*	P05655	Agriculture, food industry and synthesis
Glycogen synthase	2.4.1.21	484	100	*Triticum aestivum, Arabidopsis thaliana, Oryza sativa*	P39125	Agriculture, analysis, , biofuel production, food industry, nutrition
Maltose phosphorylase	2.4.1.8	757	100	*Levilactobacillus brevis, Plesiomonas sp., Enterococcus hirae, Emticicia oligotrophica*	O06993	Enzymatic activity analysis, Biotechnology and synthesis
Cephalosporin-C deacetylase	3.1.1.41	318	100	*B.subtilis/pumilus, Rhodotorula glutinis, Pseudomonas sp. SE83*	P94388	synthesis
Acetylxylan esterase	3.1.1.72	323	100	*B. pumilus, Streptomyces lividans, Trichoderma reesei, Penicillium chrysogenum, Aspergillus ficuum*	O31523	Enzymatic activity analysis, biofuel production, Biodegradation, medicine and synthesis
Chitosanase	3.2.1.132	277	100	*Bacillus sp., Paenibacillus sp., Microbacterium sp.*	O07921	Agriculture, enzymatic activity analysis, biotechnology, food industry, industry, medicine, pharmacology & synthesis
beta-Nacetylhexosaminidase	3.2.1.52	642	100	*A. niger/flavus/oryzae, Bacillus sp., Penicillium chrysogenum,*	P40406	Enzymatic activity analysis, diagnostic, drug development, food industry and medicine
Neopullulanase	3.2.1.135	602	61	*B.subtilis, Geobacillus stearothermophilus, Bacteroides thetaiotaomicron, Alicyclobacillus acidocaldarius*	A0A090IQN4	Enzymatic activity analysis, Biotechnology, food industry and synthesis
Levanase	3.2.1.65	516	100	*A. niger, B.subtilis/licheniformis, Lactiplantibacillus plantarum, Paenibacillus amylolyticus, Streptomyces sp.*	O07003	Agriculture, Enzymatic activity analysis, food industry, medicine and synthesis
Mannan endo-1,4beta-mannosidase	3.2.1.78	362	100	*B.subtilis, Clostridium cellulovorans, Hypothenemus hampei,*	O05512	Agriculture, Biotechnology, degradation, drug development, food industry, industry,
				Acetivibrio thermocellus, A. fumigatus		medicine, nutrition, paper production, pharmacology & synthesis

Pectin lyase	4.2.2.10	345	99.7	*Aspergillus niger*, *T. reesei or longibrachiatum*, *Alkalihalobacillus clausii*, *Penicillium italicum*	O34819	Agriculture, food industry, industry, nutrition & synthesis

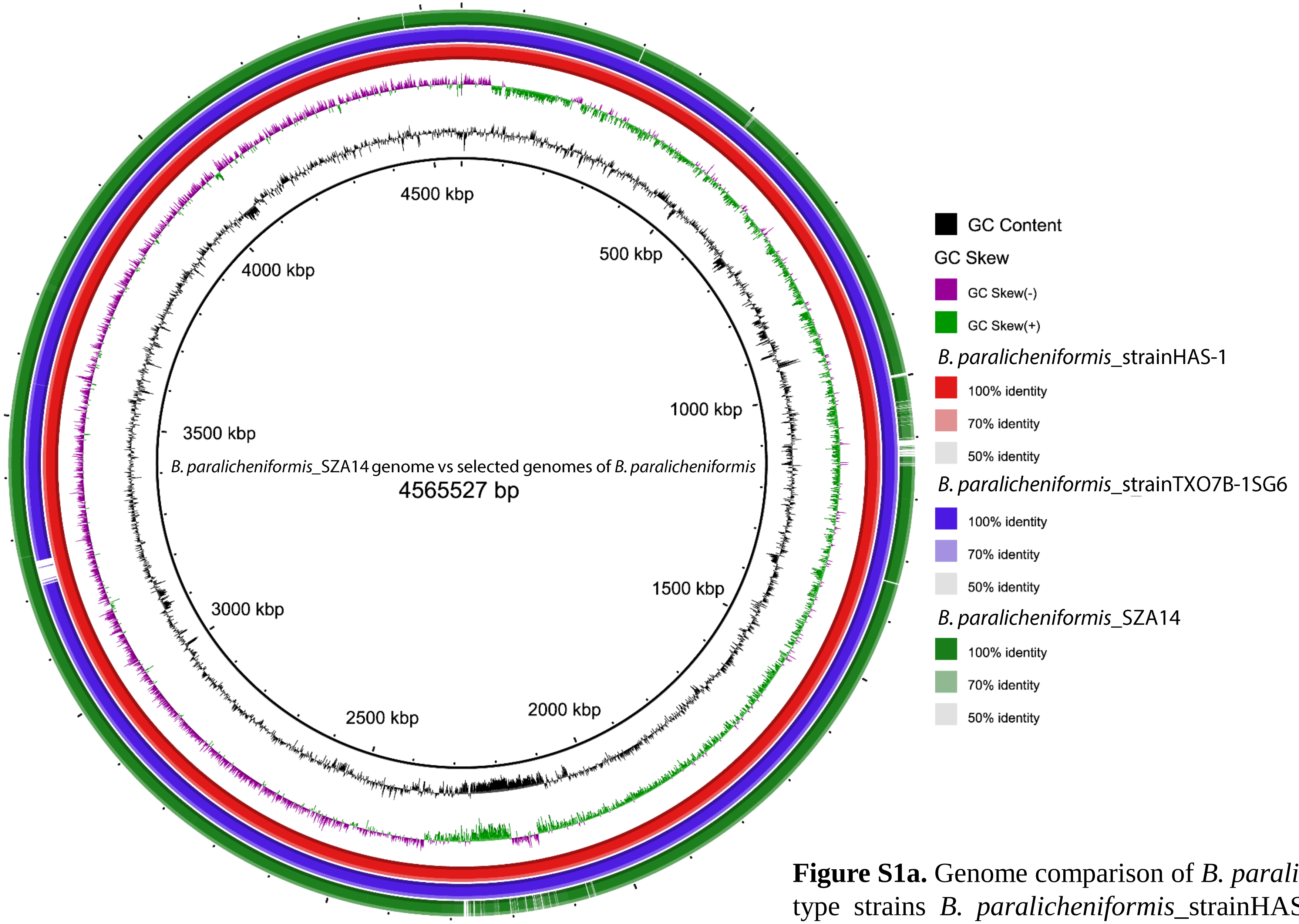

Figure S1a. Genome comparison of *B. paralicheniformis*_SZA14 against closely related *Bacillus* type strains *B. paralicheniformis*_strainHAS-1 and *B. paralicheniformis*_strainTXO7B-1SG6. From inner to outer ring: 1) GC content; black 2) GC Skew; purple-green 3) *B. paralicheniformis* strain_HAS-1 nucleotide sequence; red 4) *B. paralicheniformis*_strainTXO7b-1SG6 nucleotide sequence; Blue 5) *B. paralicheniformis*_SZA14 nucleotide sequence; Green. The circular ring map was constructed by BLAST Ring Image Generator (BRIG, version 0.95).

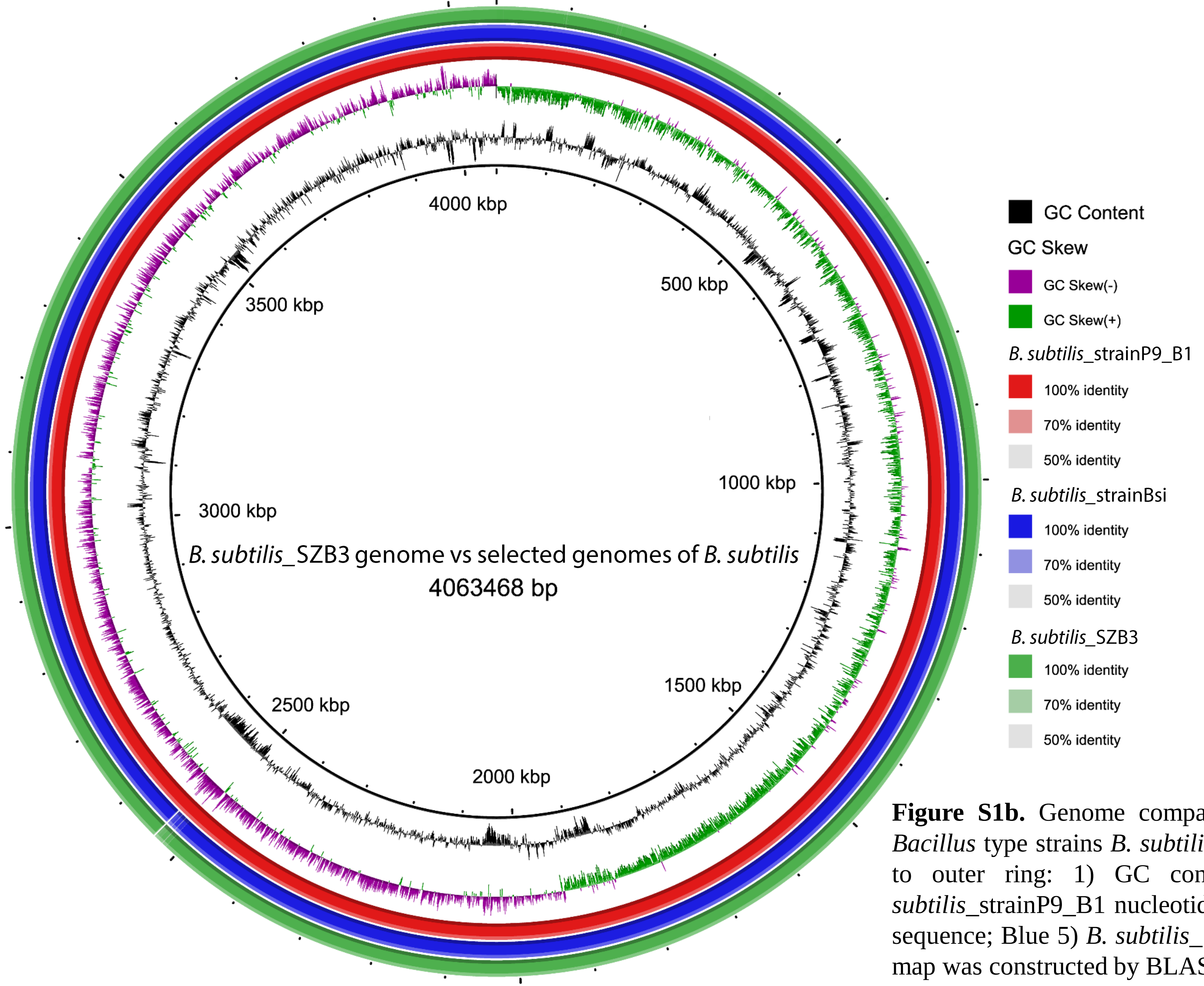

Figure S1b. Genome comparison of *B. subtilis*_SZB3 against closely related *Bacillus* type strains *B. subtilis*_strainP9_B1 and *B. subtilis*_strainBsi. From inner to outer ring: 1) GC content; black 2) GC Skew; purple-green 3) *B. subtilis*_strainP9_B1 nucleotide sequence; red 4) *B. subtilis*_strain Bsi nucleotide sequence; Blue 5) *B. subtilis*_SZB3 nucleotide sequence; Green. The circular ring map was constructed by BLAST Ring Image Generator (BRIG, version 0.95).

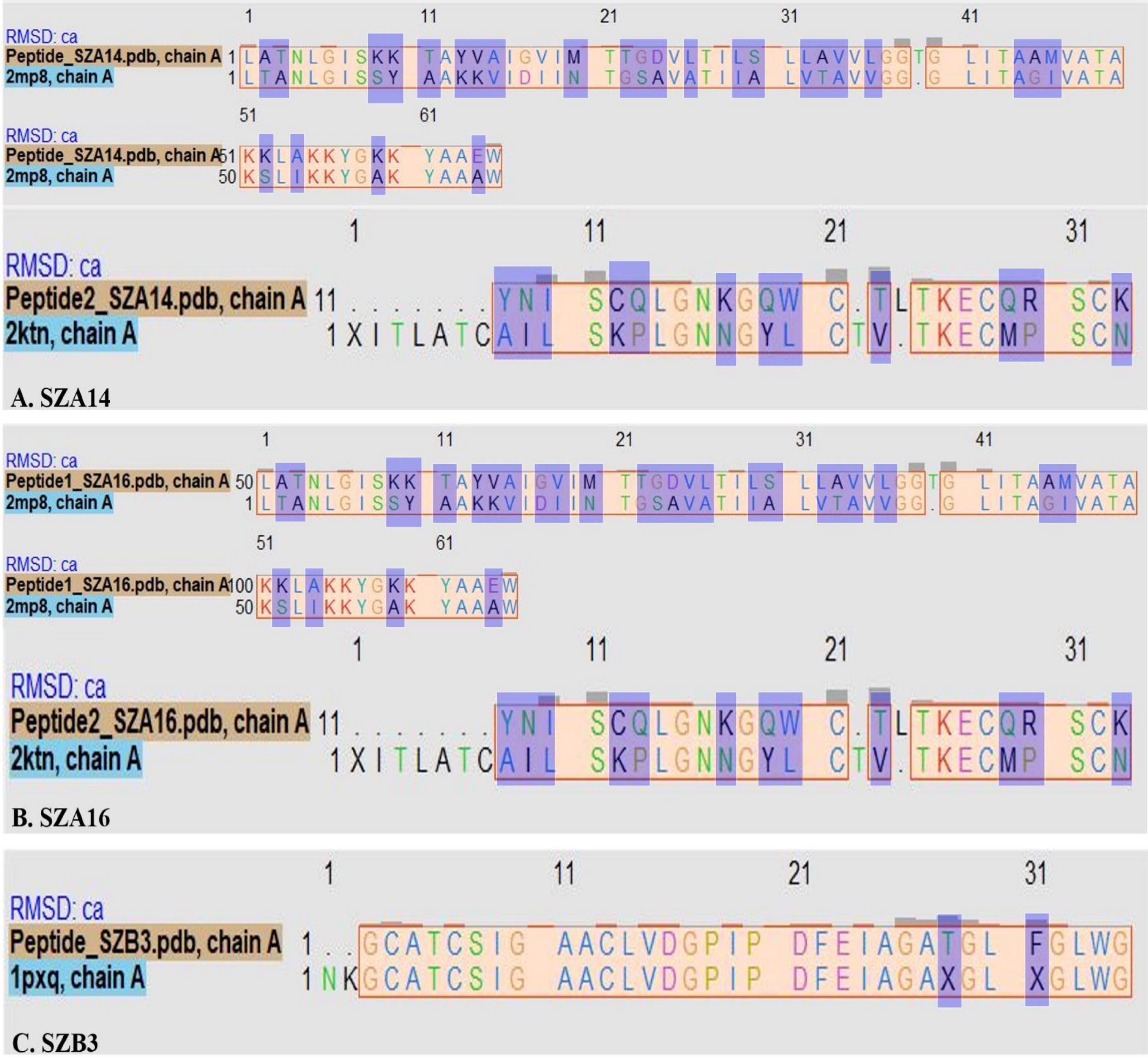

Figure S2. Multiple sequence alignment of predicted peptides with their reference sequences **A)** Sequence alignment of SZA14 peptides 1 and 2 with their reference sequences, PDB IDs 2MP8 and 2KTN, respectively. **B)** Sequence alignment of SZA16 peptides 1 and 2 with their reference sequences, PDB IDs 2MP8 and 2KTN, respectively. **C)** Sequence alignment of the SZB3 peptide with the reference sequence 1PXQ. The highlighted region shows the sequence variation. The dot (.) indicates an insertion at the specific position. The letter "X" represents dehydrated modified amino acids.
